# A crowdsensing platform for structural health monitoring of rural bridge infrastructure

**DOI:** 10.12688/openreseurope.22915.1

**Published:** 2026-02-16

**Authors:** Nikolaos D. Tantaroudas, Diego Zamora-Sanchez, Jose Alberto Armijo, Eric López-Villarragut, Ivan Arakistain, Tina Katika, Georgios Tsimiklis, Angelos Amditis

**Affiliations:** 1 Iroon Polytechneiou 9, Ethniko Metsobio Polytechneio Ereunetiko Panepistemiako Institouto Systematon Epikoinonion kai Ypologiston, Zografou, Athens, 15773, Greece; 2 Tecnalia Research & Innovation Foundation, San Sebastián, Basque Country, Spain

**Keywords:** Structural Health Monitoring, Indirect SHM, Crowdsensing, Deep Learning, Autoencoder, Internet of Things, Bridge Monitoring, Rural Infrastructure, Digital Twin, Machine Learning

## Abstract

**Background:**

Ageing bridge infrastructure in rural areas poses significant safety challenges, yet traditional structural health monitoring systems remain prohibitively expensive for regions with limited budgets. The Horizon Europe FUTURAL project developed an innovative crowdsensing platform to address this gap, enabling affordable infrastructure monitoring through vehicle-based indirect measurements combined with low-cost Internet of Things sensors.

**Methods:**

The Resilience to Shocks Smart Solution integrates multiple components: a mobile application (FUTURAPP) connecting USB-C accelerometers to smartphones for data acquisition; cloud-based processing infrastructure for secure data transmission and storage; autoencoder neural networks trained on frequency-domain acceleration data (2-15 Hz) for unsupervised anomaly detection; finite element models developed using ANSYS APDL for structural validation; and a web-based dashboard featuring Structural Health Index visualizations and citizen engagement tools. The platform was deployed and tested on two pilot bridges: a three-span concrete road bridge in Durangaldea, Spain, and the Diakofti Bridge in Kythira, Greece.

**Results:**

The autoencoder algorithms successfully distinguished between healthy structural responses and anomalous conditions using reconstruction error as the damage-sensitive feature, with the 99th percentile threshold effectively discriminating normal from abnormal measurements. Finite element models showed good agreement with in-situ vibration frequency measurements. The web platform demonstrated high usability among non-technical users, with the intuitive Structural Health Index gauge enabling immediate comprehension of bridge conditions. Citizen issue tracking features facilitated community participation in infrastructure safety monitoring.

**Conclusions:**

The crowdsensing methodology offers significant advantages over traditional approaches in terms of cost, scalability, and accessibility for resource-constrained regions. The platform represents a meaningful advancement toward digitalization of rural infrastructure management, with methodology and tools providing a foundation for replication across European transport networks.

## Plain Language summary

Bridges in rural areas are essential for daily life as they connect villages, allow farmers to transport goods, and enable emergency services to reach people in need. However, many of these bridges are aging and need regular check-ups to ensure they remain safe. Traditional monitoring systems are expensive and require installing many sensors directly on each bridge, which is not practical for rural regions with limited budgets and hundreds of small bridges.

In this project we developed a more affordable solution. Instead of placing sensors on every bridge, we put a small, low-cost accelerometer (a device that measures vibrations) inside a vehicle connected to a smartphone. When the vehicle drives over a bridge, the sensor records how the bridge vibrates. These vibration patterns can reveal a healthy bridge that has a normal pattern, while a damaged bridge will show irregularities.

The collected data is sent to a cloud infrastructure or data lake, where an artificial intelligence system learns what normal bridge vibrations look like. When new measurements arrive, the AI compares them to what it learned and flags any unusual patterns that might indicate damage. Asset managers and citizens can then view results through a user-friendly web platform that shows in real-time the health status of the bridges. This system is tested on two real bridges: one in the Basque Country of Spain and another on the Greek island of Kythira. Both bridges are critical for their local communities and face risks from earthquakes, heavy rainfall, and floods. Our tests showed that the system can successfully distinguish between normal conditions and potential problems, offering a practical way for rural communities to monitor their bridges affordably, helping keep roads safe without requiring expensive equipment on every structure.

## Introduction

The maintenance of ageing infrastructure is taking large parts of the total budget available to transport network owners across Europe. ^1^ The continuously growing stock of bridges is getting old and many have exceeded their design service life. ^2^ To ensure the safe operation of these bridges, monitoring and continuous assessment is essential. In recent years, structural health monitoring (SHM) strategies have evolved from manual inspection to sensors-based monitoring systems. ^3, 4^ Sensor-based monitoring solutions require the direct installation of multiple sensing instruments on bridges and the analysis of the collected data. ^5^ The collected information from sensors is analysed through physics and/or data-driven techniques to extract useful features for damage detection. ^6^

However, traditional SHM methods generally require multiple sensors installed on the bridge, which increases installation and maintenance costs of the monitoring system. ^7^ In addition, it is challenging to effectively utilise the large data sets generated daily for each bridge. ^8^ Because of these practical and economic considerations, the implementation of such systems is generally limited to a relatively small amount of long-span bridges, leaving the vast majority of rural bridge infrastructure unmonitored. ^9^

Rural areas face particular challenges in infrastructure monitoring due to limited budgets, sparse populations, and the dispersed nature of critical transport nodes. ^10^ Bridges in rural settings often serve as essential connectivity links, and their failure can have disproportionate impacts on local communities, including isolation of villages, disruption of agricultural supply chains, and delayed emergency response times. ^11^ Despite their importance, rural bridges typically receive less attention in terms of monitoring investments compared to major urban infrastructure. ^12^

The emergence of the Internet of Things (IoT) and advances in low-cost sensing technology have opened new possibilities for widespread infrastructure monitoring. Simultaneously, developments in machine learning and deep learning have enabled more sophisticated analysis of monitoring data, potentially allowing for automated anomaly detection without requiring extensive domain expertise. ^13^ These technological advances, combined with the increasing availability of connected vehicles, suggest that new paradigms for infrastructure monitoring may be feasible.

This paper presents the Resilience to Shocks Smart Solution developed within the Horizon Europe FUTURAL project, an innovative crowdsensing platform for infrastructure health monitoring tailored to address the challenges of rural areas. The platform aims to ensure the safety of critical infrastructure, particularly bridges in rural settings, by integrating low-cost IoT accelerometers on vehicles with additional sensors positioned directly on the infrastructure and weather station data. The primary objective is to enable the assessment of bridges before, during, and after climatic or natural phenomena (shocks), utilising a combination of indirect anomaly detection through a crowdsensing approach with vehicles and direct damage detection with strategically placed, cost-optimised sensors.

The remainder of this paper is organized as follows: Section 2 presents an extensive literature review covering the evolution of indirect SHM methods and machine learning applications in bridge monitoring. Section 3 describes the materials and methods, including the system architecture, data acquisition approach, AI algorithms. Section 4 presents the pilot case descriptions. Section 5 presents the results of the autoencoder training and validation, the finite element modeling, the stakeholder engagement and co-creation and the platform implementation and deployment. Section 6 discusses the findings and their implications, and Section 7 concludes the paper with future directions.

## Literature review

### Evolution of bridge structural health monitoring

Structural health monitoring has evolved significantly over the past decades, transitioning from periodic manual inspections to continuous automated monitoring systems. ^14^ Traditional approaches rely on visual inspections, which are subjective, time-consuming, and may fail to detect internal damage or damage in inaccessible locations. The development of sensor technologies enabled direct monitoring approaches, where instruments such as accelerometers, strain gauges, and displacement sensors are installed directly on the structure. ^15, 16^ Direct SHM systems have been successfully deployed on major bridges worldwide, including long-span bridges and critical infrastructure assets. ^17^ These systems typically employ networks of sensors connected to data acquisition systems, with analysis performed either on-site or in remote monitoring centres. ^18^ However, past studies have shown that the cost of installation, maintenance, and data management for direct monitoring systems limits their deployment to high-value structures, leaving the vast majority of the bridge stock unmonitored. ^2^

### Indirect structural health monitoring

As an alternative to traditional SHM methods, many studies have proposed indirect or 'drive-by' methods that utilise the measured responses from a moving vehicle while traversing the bridge of interest. ^19^ The method was initially proposed by Yang et al. ^20^ in 2004 to identify the bridge's natural frequencies from the dynamic response of a passing vehicle. This preliminary work demonstrated that bridge dynamic properties could be extracted from vehicle acceleration signals, opening a new research direction that has attracted considerable attention over the past two decades. The theoretical foundation of indirect SHM rests on the vehicle-bridge interaction (VBI) phenomenon, where the dynamic response of a passing vehicle is influenced by the bridge's structural properties. ^21^ When a vehicle crosses a bridge, its vibration response contains information about both the vehicle's own dynamics and the bridge's dynamic characteristics. ^22^ By carefully analysing the vehicle's acceleration signal, it is theoretically possible to extract information about the bridge's condition. Lin and Yang ^23^ experimentally verified the feasibility of using a passing vehicle to scan the fundamental bridge frequencies, demonstrating that the concept could work in controlled conditions. Subsequent research by Yang and Chang ^24^ explored the parametric aspects of extracting bridge frequencies indirectly, identifying factors that influence the accuracy of frequency identification, including vehicle speed, bridge span length, and road surface roughness.

### Modal-based approaches

Modal-based indirect monitoring approaches focus on extracting modal parameters of the bridge, including natural frequencies, mode shapes, and damping ratios, from vehicle response signals. ^25^ Malekjafarian et al. ^26^ provided a comprehensive review of indirect bridge monitoring using passing vehicles, categorising approaches into frequency identification, mode shape extraction, and damage detection methods. The use of empirical mode decomposition (EMD) and its variants has also been explored for bridge frequency identification from drive-by measurements. Zhu and Malekjafarian ^27^ investigated the use of ensemble EMD for identifying bridge frequencies from vehicle responses, demonstrating improved performance over standard signal processing techniques. OBrien et al. ^28^ applied EMD to drive-by bridge damage detection, showing that changes in bridge condition could be reflected in the vehicle response characteristics. Mode shape extraction from indirect measurements has also been investigated. ^29^ Yang et al. ^30^ developed theoretical frameworks for constructing bridge mode shapes from passing vehicle data, while Malekjafarian and OBrien ^31^ demonstrated the use of passing vehicles for bridge mode shape estimation. More recently, Yang et al. ^32^ proposed normalised formulas for removing damping effects in recovering bridge mode shapes using moving and stationary vehicles.

### Challenges in indirect structural health monitoring

Despite significant research progress, indirect SHM remains challenging and has not yet achieved widespread practical application. ^33^ Drive-by monitoring has been mostly proposed as a pre-screening tool to detect damage under favorable conditions of low vehicle speeds and low road roughness, ^34^ with the degradation of the road profile often being indistinguishable from bridge damage. More generally, research on drive-by bridge monitoring has been ongoing for decades without reaching any level of practical application in real engineering.

Malekjafarian et al. ^26^ identified several key challenges that must be addressed: i) Short duration of signals: Vehicle crossing times are typically brief, limiting the frequency resolution available for analysis and the amount of data collected per crossing event; ii) Interference from pavement-induced vibration: Road surface roughness generates significant vibration in the vehicle that can mask the bridge-induced component of the response ^35^; iii) Environmental and operational influences: Temperature variations, traffic loading, and other environmental factors affect bridge dynamic properties, creating variability that can be mistaken for damage ^36^; iv) Quantification of damage: Even when anomalies are detected, relating them to specific damage types, locations, and severities remains difficult ^8^; v) Experimental verification: Most drive-by concepts have been demonstrated through numerical simulations or laboratory-scale experiments, with limited full-scale field validation.

Past work ^37^ has conducted investigations to identify bridge eigenfrequencies by indirect measurement, testing with different vehicle speeds, traffic and road profile conditions. They found that the first eigenfrequency could not be extracted in their experiment, while the second natural frequency was identified only under certain conditions. This failure was attributed to the high passing speeds of the cars, the absence of significant bridge excitation and the interference generated by driving on the road. In general, this research concluded that eigenfrequency detection improves when bridge spans are longer, vehicle speeds are lower and roads are better paved.

### Machine learning and deep learning for SHM

The application of machine learning (ML) and deep learning (DL) techniques to SHM has grown rapidly in recent years. ^38^ Avci et al. ^39^ provided a comprehensive review of vibration-based damage detection methods, tracing the evolution from traditional statistical approaches to modern deep learning applications. They noted that DL models have attracted attention due to their ability to automatically learn features from raw data without requiring manual feature engineering. Hinton and Salakhutdinov ^40^ introduced the concept of deep autoencoders for dimensionality reduction, demonstrating that neural networks with multiple hidden layers could learn compact representations of complex data. This work laid the foundation for using autoencoders in anomaly detection applications, including SHM. For direct SHM applications, several researchers have employed deep learning for damage detection. Abdeljaber et al. ^41^ proposed applying 1D convolutional neural networks (CNNs) to extract structural damage features from vibration response time histories. Ni et al. ^8^ used 1D CNN for data compression and anomaly detection in acceleration data from a long-span suspension bridge. Wang and Cha ^42^ combined deep autoencoders with one-class support vector machines for structural damage detection, demonstrating the potential of hybrid approaches.

### Data-driven approaches for indirect monitoring

The integration of machine learning with indirect monitoring represents a promising research direction. ^43^ Malekjafarian et al. ^44^ introduced a machine learning approach to bridge damage detection using responses measured on a passing vehicle, employing artificial neural networks to predict vehicle responses and using prediction quality as a damage indicator. This approach was later extended to account for temperature variations, demonstrating improved robustness to environmental influences. ^45^ Sarwar and Cantero ^46^ proposed a deep autoencoder architecture specifically designed for bridge damage assessment using responses from several vehicles. Their approach used 1D CNN combined with Long Short-Term Memory (LSTM) layers to extract features from vehicle acceleration signals. This work demonstrated that unsupervised learning approaches could detect bridge damage without requiring labelled damaged data, which is typically unavailable in real-world scenarios. Fernandez-Navamuel et al. ^47^ extended autoencoder-based approaches to multi-sensor data, combining local variables (inclinations and stresses) with global variables (eigenfrequencies) for damage detection. Their results revealed synergies between different variable types, achieving high classification precision for various damage scenarios. Li et al. ^48^ investigated real-time drive-by bridge damage detection using deep autoencoders with frequency-domain vehicle responses as input features, demonstrating that frequency domain representation have the potential to enhance robustness to measurement noise compared to time-domain approaches.

### Crowdsensing and fleet-based monitoring

The concept of crowdsensing for infrastructure monitoring leverages data from multiple vehicles to overcome limitations of single-vehicle approaches. Matarazzo et al. ^49^ demonstrated the feasibility of crowdsourcing bridge dynamic monitoring using smartphone vehicle trips, showing that aggregate data from multiple passages could provide meaningful structural information. Gkoumas et al. ^50^ summarised past studies on indirect SHM, identifying three key points: (i) iSHM with a single vehicle is difficult; (ii) direct monitoring with a fleet of vehicles is feasible; and (iii) iSHM with a fleet of vehicles represents the future direction for the field. This represents an innovative case from the current situation where most monitoring is by human inspection to a future where most monitoring will be indirect, with instrumentation in passing vehicles. OBrien ^51^ proposed mining data from fleets of instrumented passing vehicles, describing a process involving: (a) processing accelerations using inverse Newmark-beta computational methods to determine moving reference deflections; (b) using off-bridge data to calibrate fleet vehicles; and (c) using on-bridge data to determine moving-reference influence lines as damage-sensitive features.

### IoT and low-cost sensing for rural applications

The development of low-cost IoT sensors has opened possibilities for widespread monitoring deployment. ^52^ Peng et al. ^53^ developed and experimentally verified an IoT sensing system specifically designed for drive-by bridge health monitoring, demonstrating that affordable sensor technology could provide adequate data quality for damage detection applications. In addition, previous study by Garcia-Sanchez et al. ^54^ presented the crowdsensing approach developed within the FUTURAL project, combining accelerations measured by onboard sensors with different bridge and weather event measurements to drive a cost-effective solution for structural assessment. These studies highlighted that the approach relies on multi-sensor information and proposes a vehicle-only methodology complemented by direct SHM approaches.

## Methods and materials

### System architecture overview

The Resilience to Shocks Smart Solution is a novel cellular IoT sensor data cloud monitoring system designed to enhance the resilience of infrastructure, specifically in rural areas where budget constraints often limit the deployment of advanced monitoring technologies. ^55^ The system utilises low-cost IoT sensors to gather data for structural health monitoring and optimises it by employing both direct and indirect monitoring techniques.

The architecture comprises several interconnected components: i) Data acquisition through FUTURAPP mobile application and IoT sensors; ii) Intermediate cloud processing infrastructure; iii) AI/ML algorithms for anomaly detection; iv) Finite element models for structural validation; v) A crowdsensing platform including a web-based dashboard for visualisation and user interaction. These components work together to process and examine the data gathered from various sources, such as vehicles and on-site sensors, offering real-time insights into the health condition of the infrastructures. The system utilises AI and ML algorithms to analyse data acquired by MEMS sensors embedded in low-cost IoT devices.

**Figure 1.**
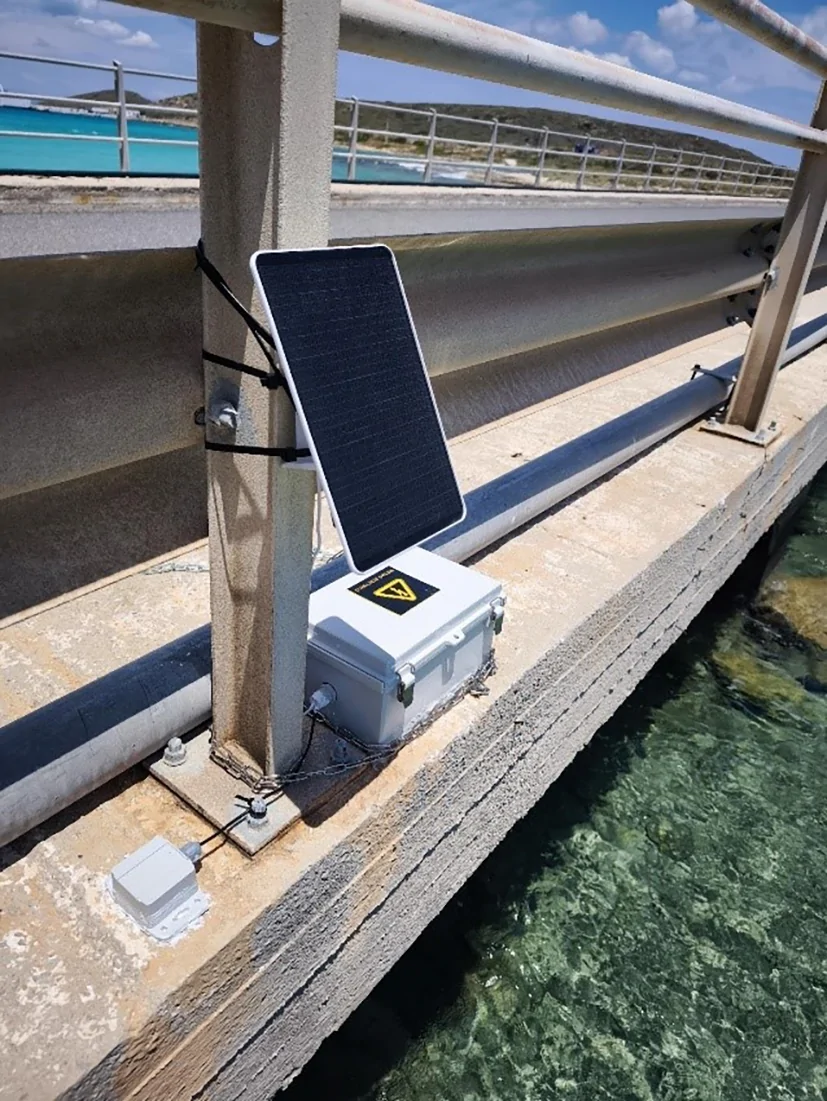
IoT low-budget sensor installation including solar panel for power provision, accelerometer and hardware.

Figure 1 shows the on-site IoT sensor installation on the Diakofti Bridge in Kythira, Greece. The direct monitoring station comprises a solar panel for autonomous power supply, a weatherproof enclosure housing the data acquisition and wireless transmission electronics, and a compact accelerometer (gray sensor, bottom left) mounted directly on the bridge deck to capture structural vibrations. The self-sustaining design eliminates the need for external power connections, making it suitable for remote rural infrastructure in aggressive marine environments. This direct monitoring approach complements the indirect vehicle-based crowdsensing methodology, providing continuous on-structure measurements for enhanced anomaly detection capabilities.

An intermediate cloud architecture acts as a bridge between raw data collection from the infrastructure asset and the crowdsensing platform. This intermediate infrastructure sends raw data to a selected IP address for initial collection and analysis, dispatches data/alerts/evaluations to facilitate timely responses to detected anomalies, and provides a direct link to with dashboards and indicators of the specific pilot use case.

### Data acquisition: The FUTURAPP mobile application

**Figure 2.**
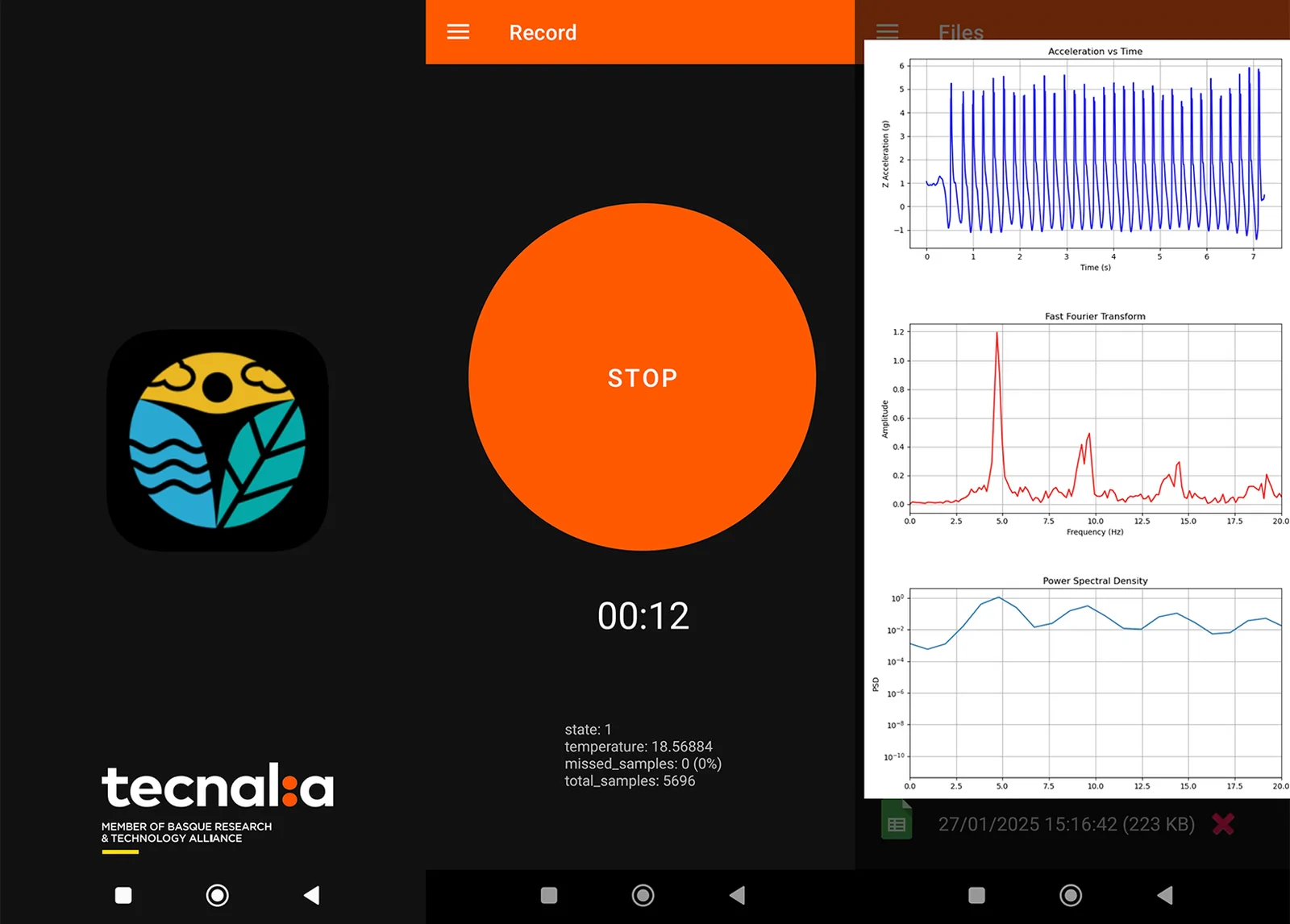
Sample images from FuturAPP. Left. Automatic pop-up when the sensor is connected to the device (tablet or smartphone). Centre. Main panel that allows to configure the measurement and start/stop it. Right. Results of automatic processing of acceleration.

At the core of the data collection process is the FUTURAPP application developed by TECNALIA within the FUTURAL project. ^55^ This mobile application allows connecting a USB-C accelerometer (specifically, the PLab PicoQuake sensor) with high specifications and low price to a tablet or smartphone ( Figure 2).

The main features of FUTURAPP include: i) Automatic recognition of PLab's PicoQuake sensor when connected to the smartphone's USB-C port; ii) Application menu for setup, start, and stop of measurements; iii) Automatic saving of completed measurements; iv) File viewing organised by date and time; v) Automatic Fast Fourier Transform (FFT) processing for each acceleration record; vi) Data communication capabilities for transmitting measurements to the cloud platform. Data collection involves monitoring accelerometers recording acceleration values over time. These raw datasets are then transformed into the frequency domain for detailed analyses.

### Cloud infrastructure and data management

Data storage and export processes primarily utilise CSV files with conversions to Excel, ASCII, or other formats as required. The default communication method involves using JSON messages or data packets, which are sent through SigFox or 3G/4G and MQTT protocol to a broker. From the intermediate broker, these messages are forwarded to either a cloud server or a data lake for storage in the cloud. Specific security measures are in place to protect the collected data, including: i) Database backup systems; ii) Standard IoT protocols for secure data transmission; iii) SSL certificates on the web platform for data privacy protection.

### Autoencoder neural network for anomaly detection

The system employs autoencoder neural networks for anomaly detection based on the reconstruction error principle. The architecture follows the typical structure of an autoencoder (encoder, code and decoder), reducing the number of nodes in the hidden layers to constrain the flow of information through the network. This approach enables the model to learn the most important or characteristic attributes of the input data and reconstruct them optimally.

The autoencoder consists of an encoder, which can be mathematically defined as:

f(x)=h

Where x is the input data, and h are the latent variables in the bottleneck layer. The decoder takes these latent variables and maps them to an output, which can be defined as:

$$g\left(h\right)=x^{\prime }$$

The whole autoencoder can be defined as:

L(x,g(f(x)))

where L represents the loss function measuring the reconstruction error between the input x and the reconstructed output g(f(x)). The proposed autoencoder architecture is detailed in Table 1, showing the encoder configuration with three fully connected layers using ReLU activation and the mirrored decoder structure with sigmoid output activation.

**Table 1.** Proposed architecture for encoder and decoder.

Encoder layer	Output shape	Activation	Decoder layer	Output shape	Activation
Input	(650)		Dense	(32)	ReLU
Dense	(300)	ReLU	Dense	(128)	ReLU
Dense	(64)	ReLU	Dense	(300)	ReLU
Dense	(16)	ReLU	Output	(650)	Sigmoid

**Figure 3.**
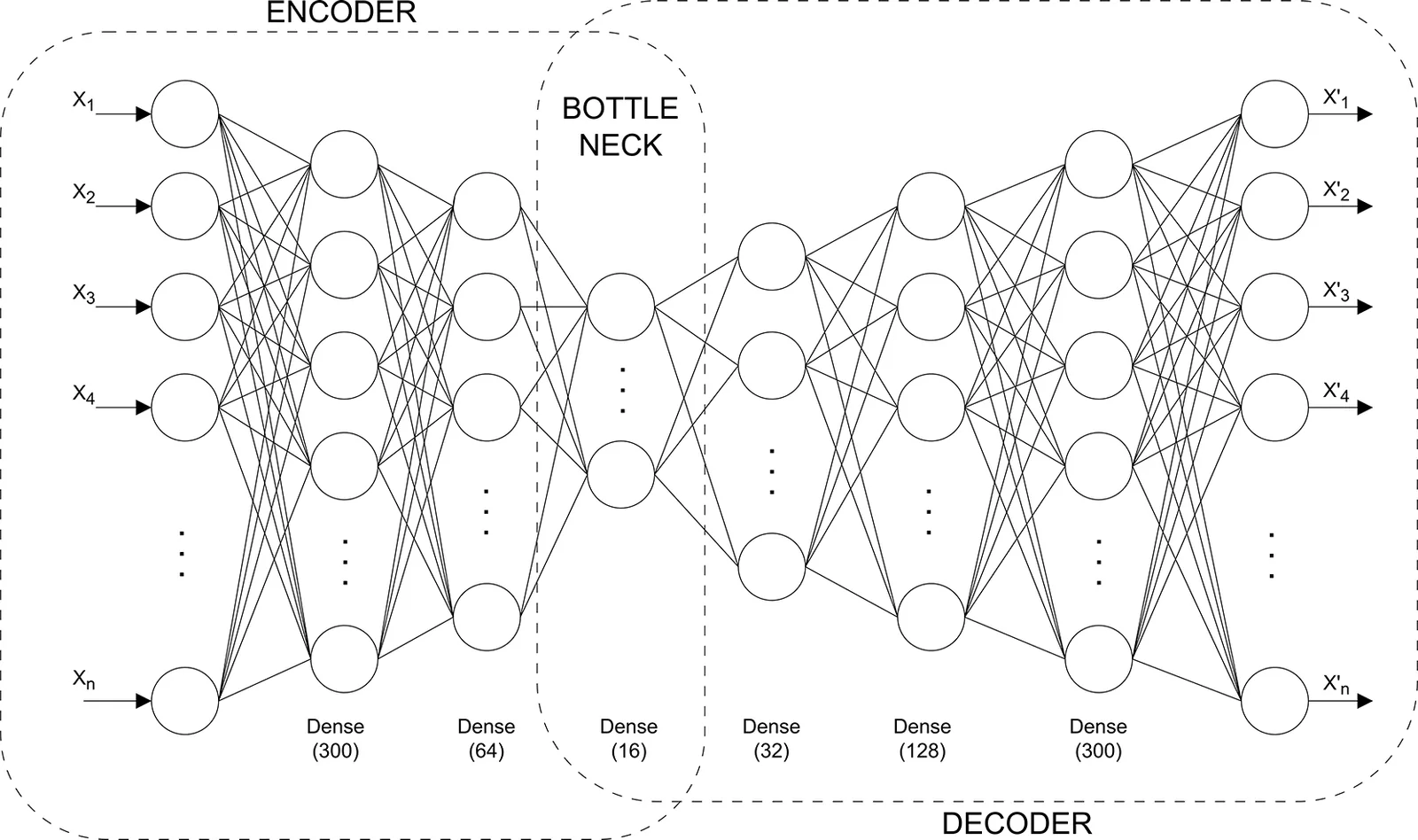
Scheme of network architecture for the proposed autoencoder.

The encoder consists of one input layer and three fully connected layers with ReLU (Rectified Linear Unit) as activation function. For the decoder, the same architecture is used but in a mirrored fashion, with another three fully connected layers and the output layer with a sigmoid as activation function. Figure 3 illustrates the scheme of the network architecture for the proposed autoencoder used in this study.

## Pilot case descriptions

### Durangaldea, Spain

**Figure 4.**
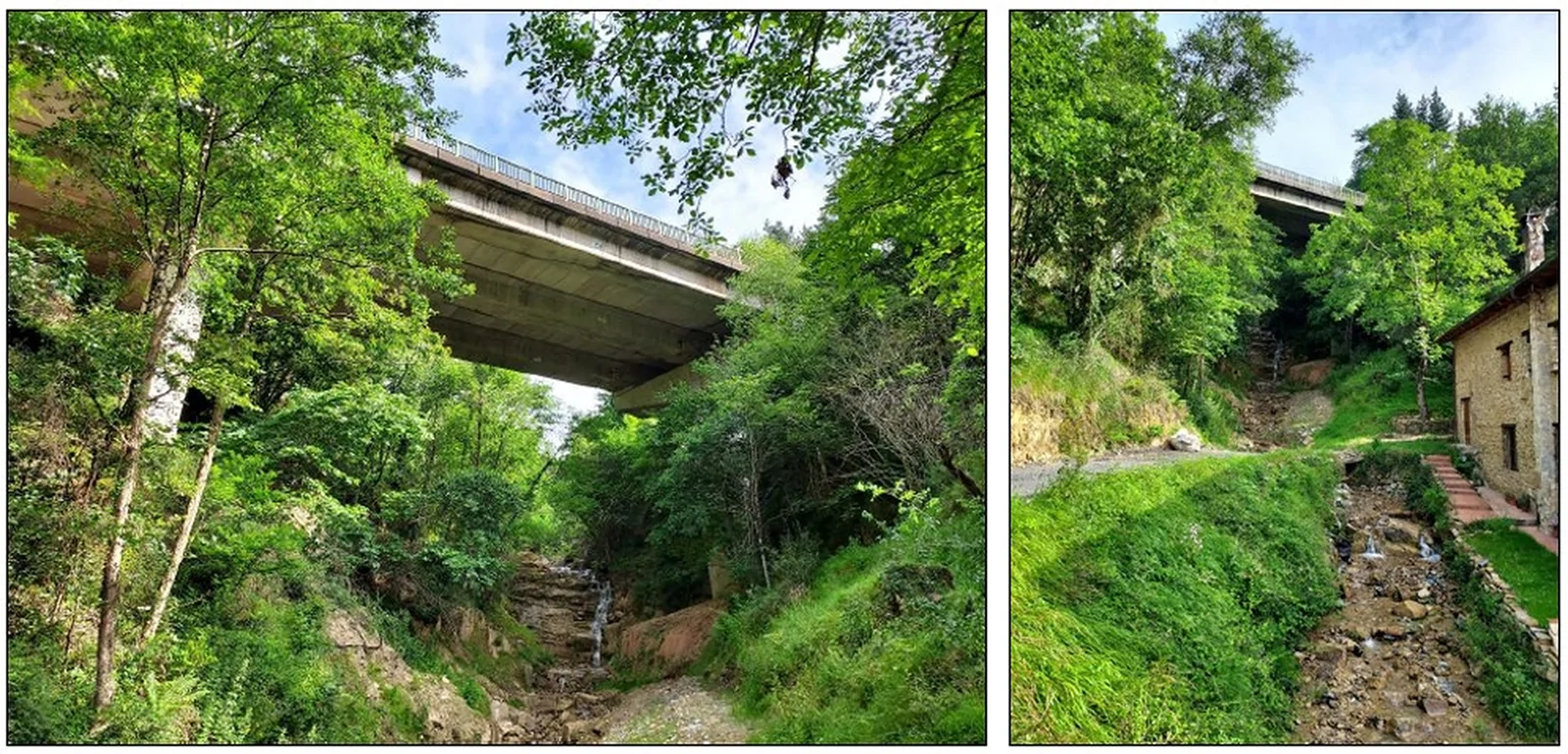
Durangaldea pilot case bridge and surroundings.

**Figure 5.**
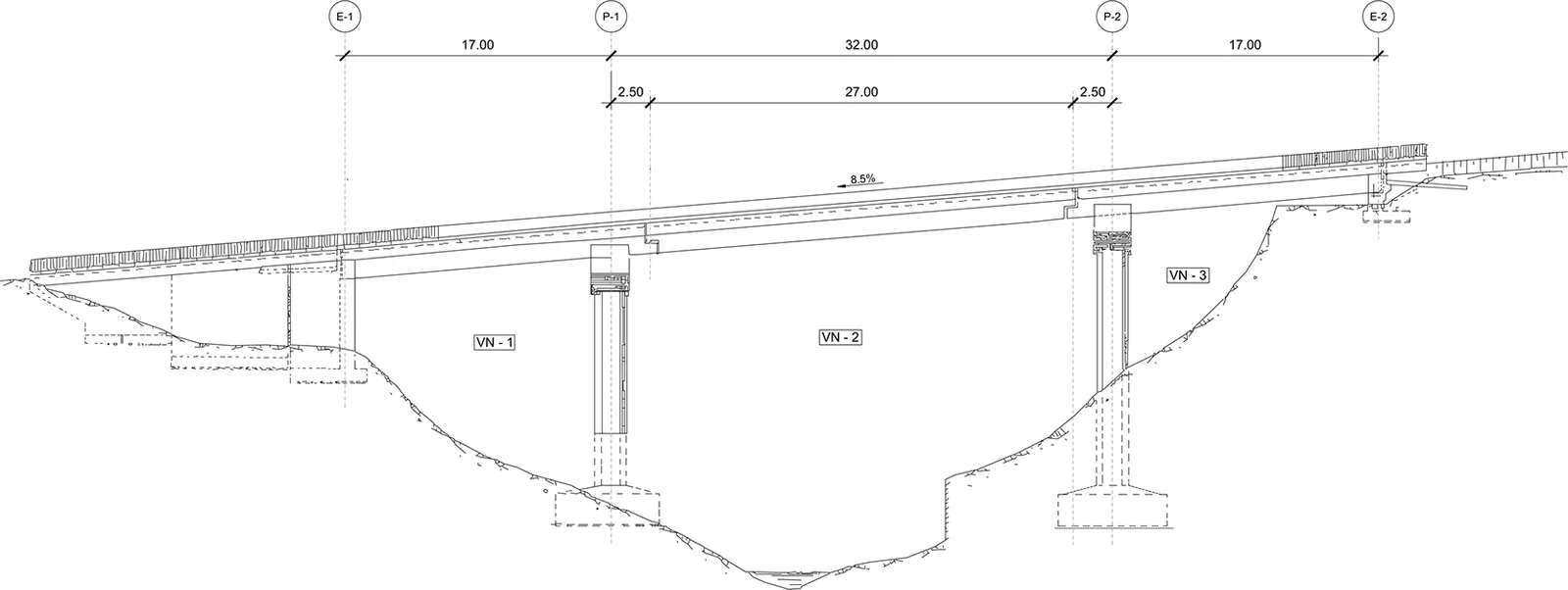
Bridge Drawing showing its profile and length of the spans. Image provided by the Provincial Council of Bizkaia (Public Administrator of this infrastructure in Durangaldea).

The Durangaldea pilot case focuses on a critical transportation node for this rural area consisting of a 3-span concrete road bridge with high piers, and an important central isostatic span resting on half-joint (or dapped-end) supports ( Figure 4 and Figure 5). The bridge is located on a main road essential for regional connectivity and is particularly vulnerable to environmental risks such as heavy rainfall and slope instability, with the area beneath the piers exposed to torrential watercourses and debris flow. ^55^

Interactions with stakeholders, particularly the Provincial Council of Bizkaia (Public Administrator of this infrastructure), have been useful in refining the system's focus, highlighting the importance of usability, scalability, and feasibility for broader applications across rural areas with similar vulnerabilities.

### Kythira, Greece

**Figure 6.**
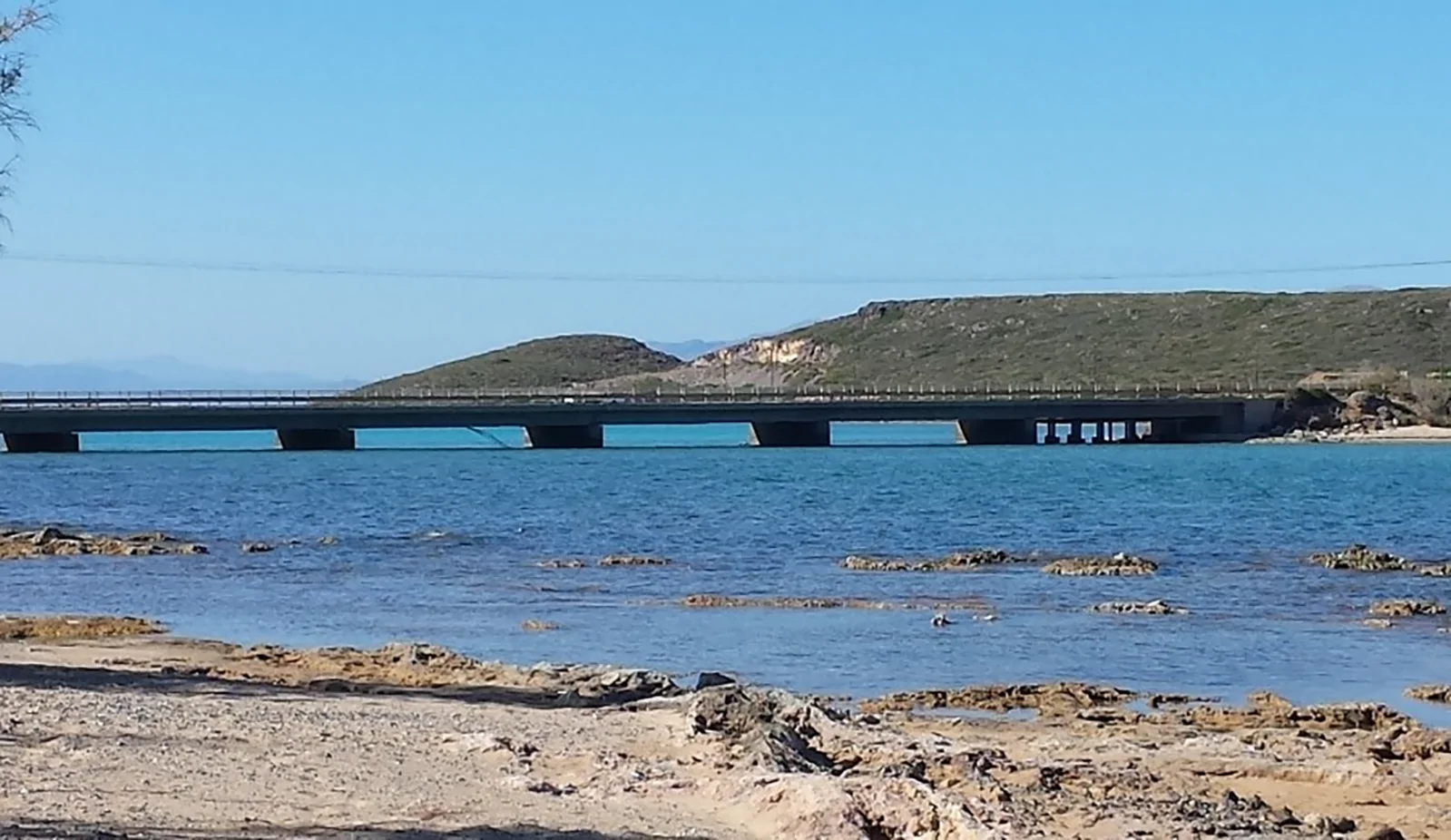
Kythira pilot case bridge.

The Kythira pilot case focuses on the Diakofti Bridge, an iconic structure on the island that provides access to vehicles from ferries arriving on the island ( Figure 6). The bridge is located in an aggressive marine environment close to the port and is vulnerable to shocks such as earthquakes, heavy rainfall, and floods, which can compromise both structural integrity and transportation networks. ^55^

The Resilience to Shocks Smart Solution was deployed at this location to enable early detection of anomalies and damage, ensuring swift responses to potential risks and promoting the long-term safety and sustainability of this critical transport route on the island.

## Results

### Autoencoder training and validation

**Figure 7.**
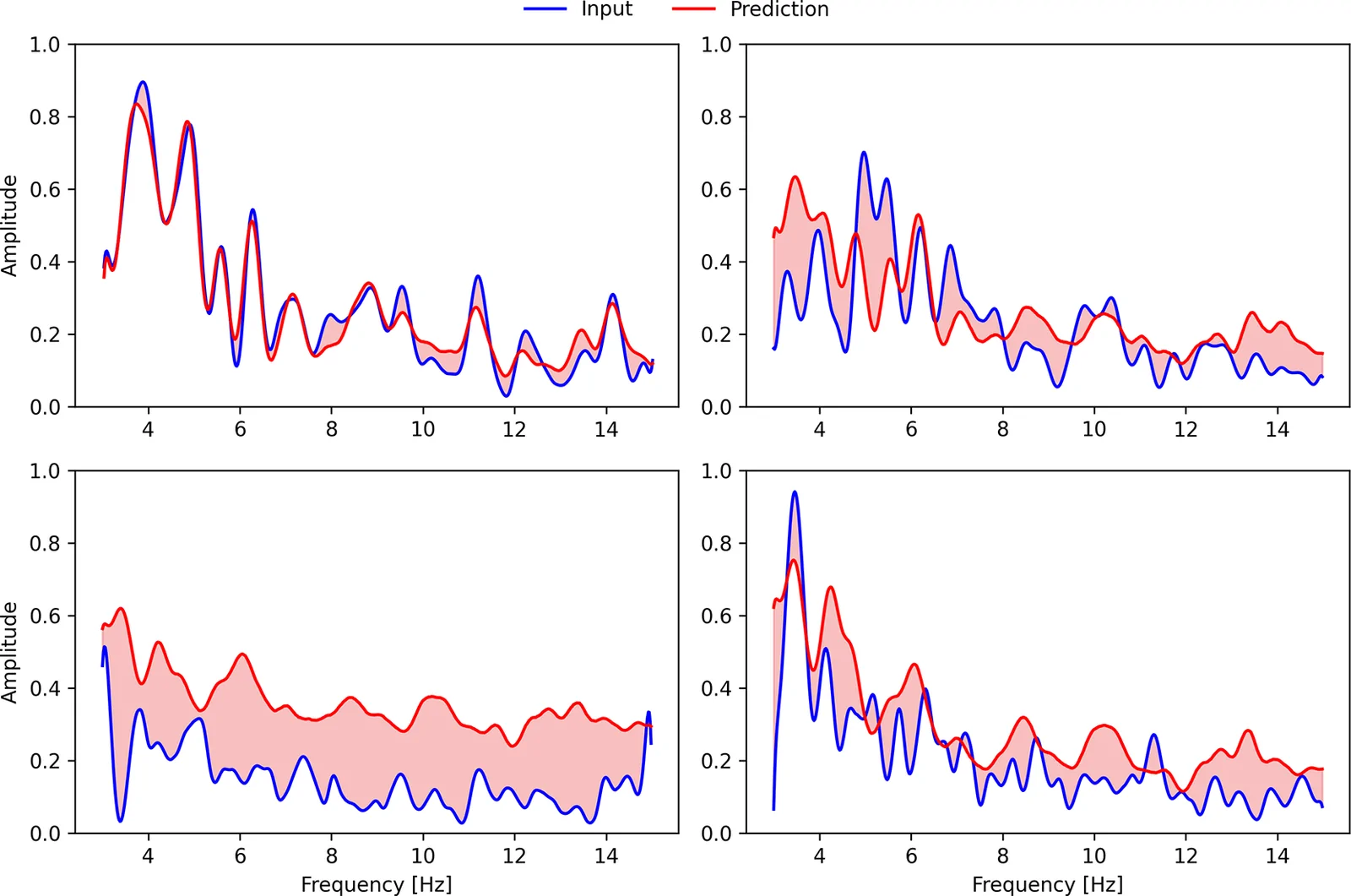
Reconstruction for a normal signal (top left) and abnormal test signals.

As training data, a dataset prepared with 30 passes of the car over the pilot bridge have been used. The accelerations obtained in these 30 passes have been transformed to the frequency domain and clipped to the frequency spectrum of 2-15Hz, and their magnitude or amplitude normalised to values from 0 to 1. The reconstruction error (the difference between the original input signal and the reconstructed output) serves as the damage-sensitive feature. A damage threshold is set at the 99th percentile of reconstruction errors from the training signals, which are considered normal given prior analysis and selection. ^55^ For the testing phase, unseen passes of the vehicle over the bridge are classified as normal (when below the threshold), while contrast cases (such as flat road signals) are clearly identified as abnormal based on their larger reconstruction error area. Given the enormous difficulty of iSHM approaches, this study proposes a vehicle-only approach that is complemented by other direct SHM approaches. Figure 7 illustrates the reconstruction for a normal signal (top left) and abnormal test signals. The error area between the original input and reconstructed output is shown filled in red. The normal bridge crossing signal exhibits minimal reconstruction error, while the abnormal test signals (flat road passages) display significantly larger error areas, demonstrating the autoencoder's ability to discriminate between healthy and anomalous conditions.

**Figure 8.**
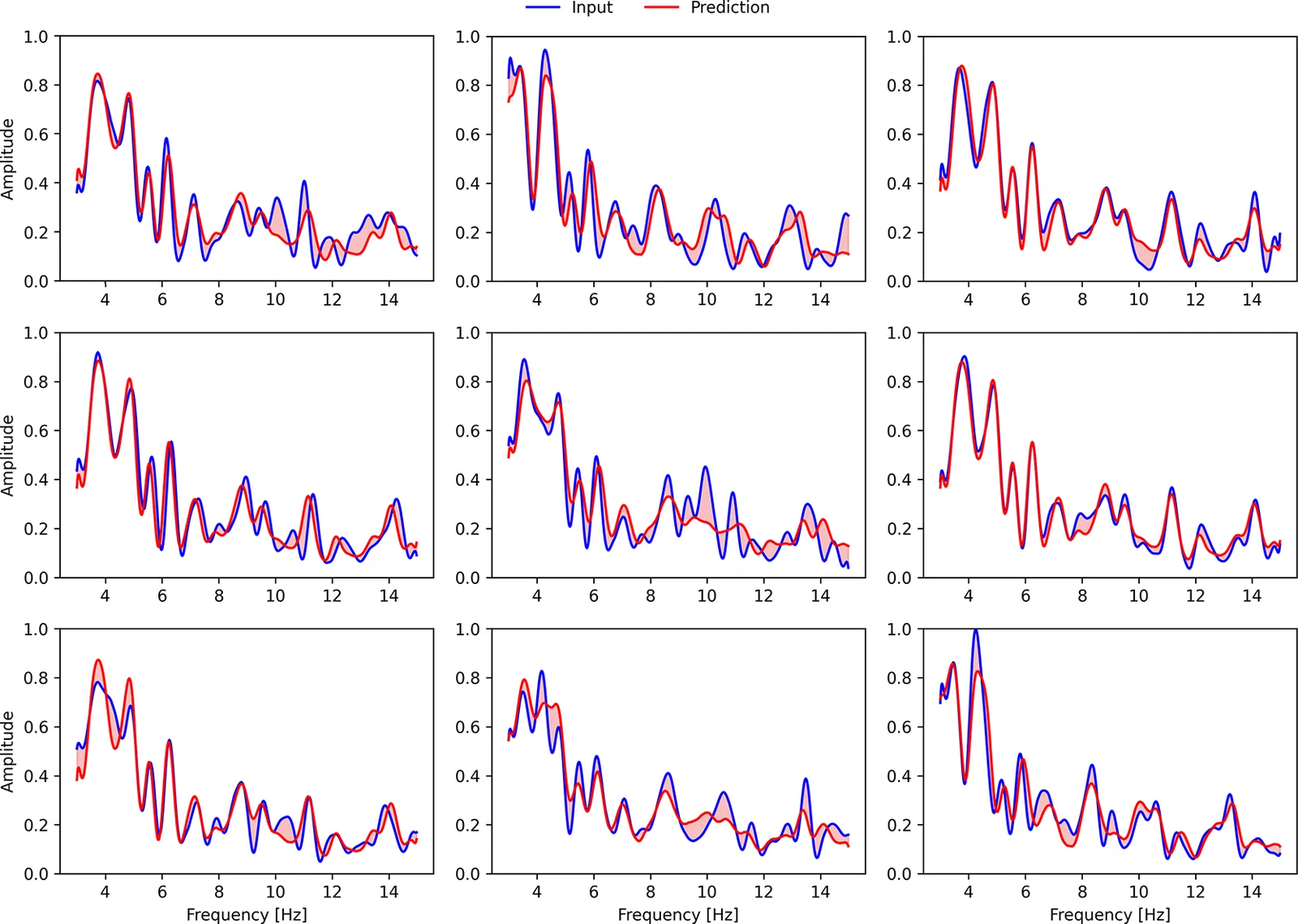
Reconstruction for different car passages in the training dataset.

The reconstruction error of the Autoencoder has been found to be relatively small. Training samples that are found normal have been considered, given the short time elapsed and after prior analysis and selection, and set a damage threshold for which above the 99th percentile of these training signals is considered normal. Then, for the testing phase unseen passes of the vehicle over the bridge are classified as normal, while other (simple) contrast cases are clearly identified as abnormal. The results demonstrate that: i) normal bridge crossing signals produce small reconstruction errors; ii) abnormal signals (such as flat road passages) produce much larger reconstruction error areas; iii) The 99 ^th^ percentile threshold effectively discriminates between normal and abnormal conditions. Figure 8 illustrates the reconstruction results for different car passages in the training dataset. The small reconstruction errors (shown in red shading) across multiple vehicle crossings indicate that the autoencoder has successfully learned to represent the characteristic frequency-domain patterns of healthy bridge responses during the training phase.

### Finite element modeling

To validate the structural integrity and behavior under various conditions and ensure the system's reliability and effectiveness, computational finite element (FE) models were developed using ANSYS APDL parametric scripting. ^56^ A parametric ANSYS APDL script has been developed to simulate the vibration behaviour of the pilot bridge, showing good agreement with the eigenfrequencies extracted from a high-end environmental vibration accelerometer (GeoSIG AC-73). ^55^

**Figure 9.**
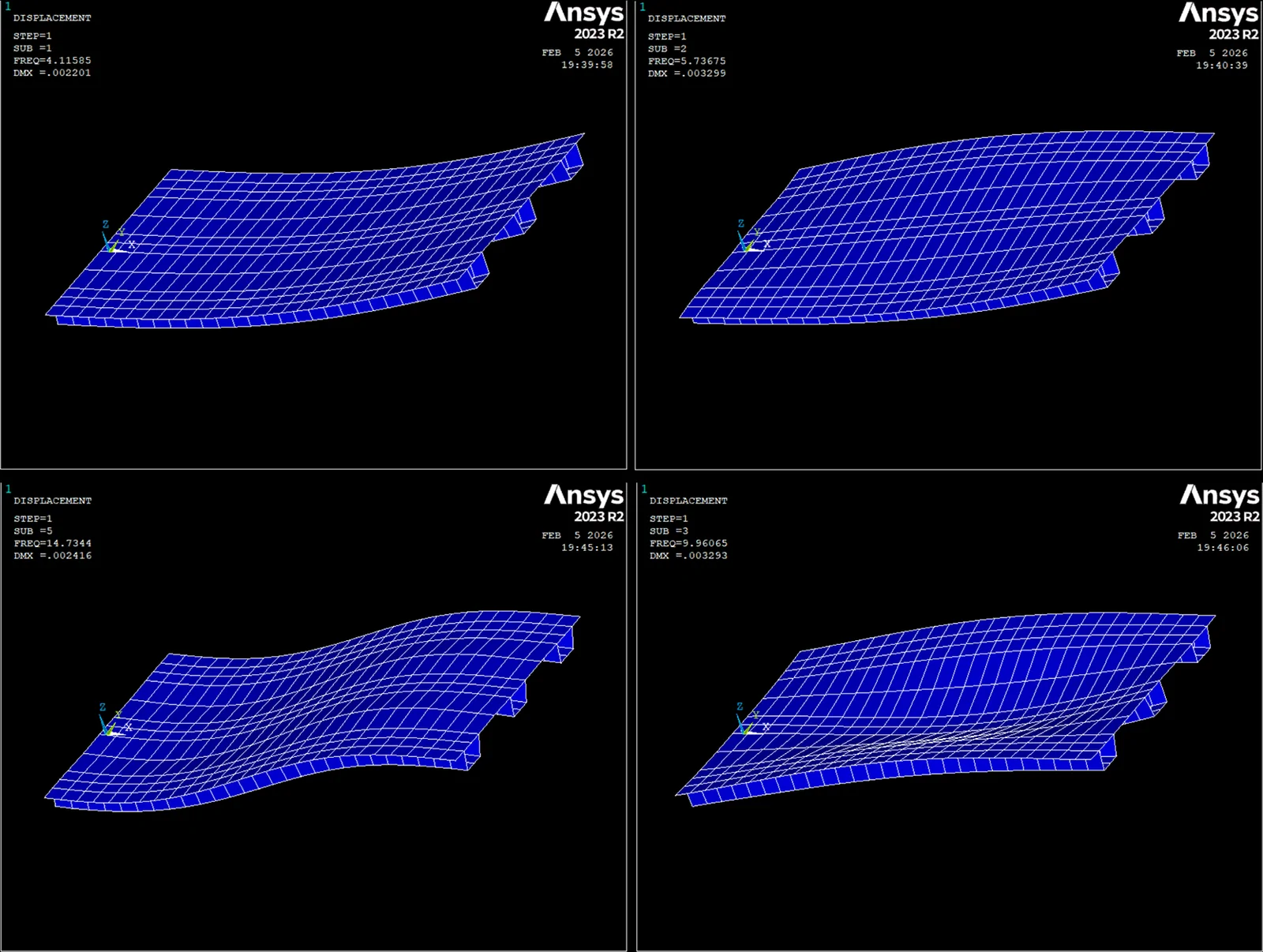
Finite element model for the central span of pilot bridge in Durangaldea. The bridge on BI-633 road in Berriz (rural area of Durangaldea, Bizkaia, Spain) is a 3-span road bridge with high piers, and a long central span resting on half-joint.

**Figure 10.**
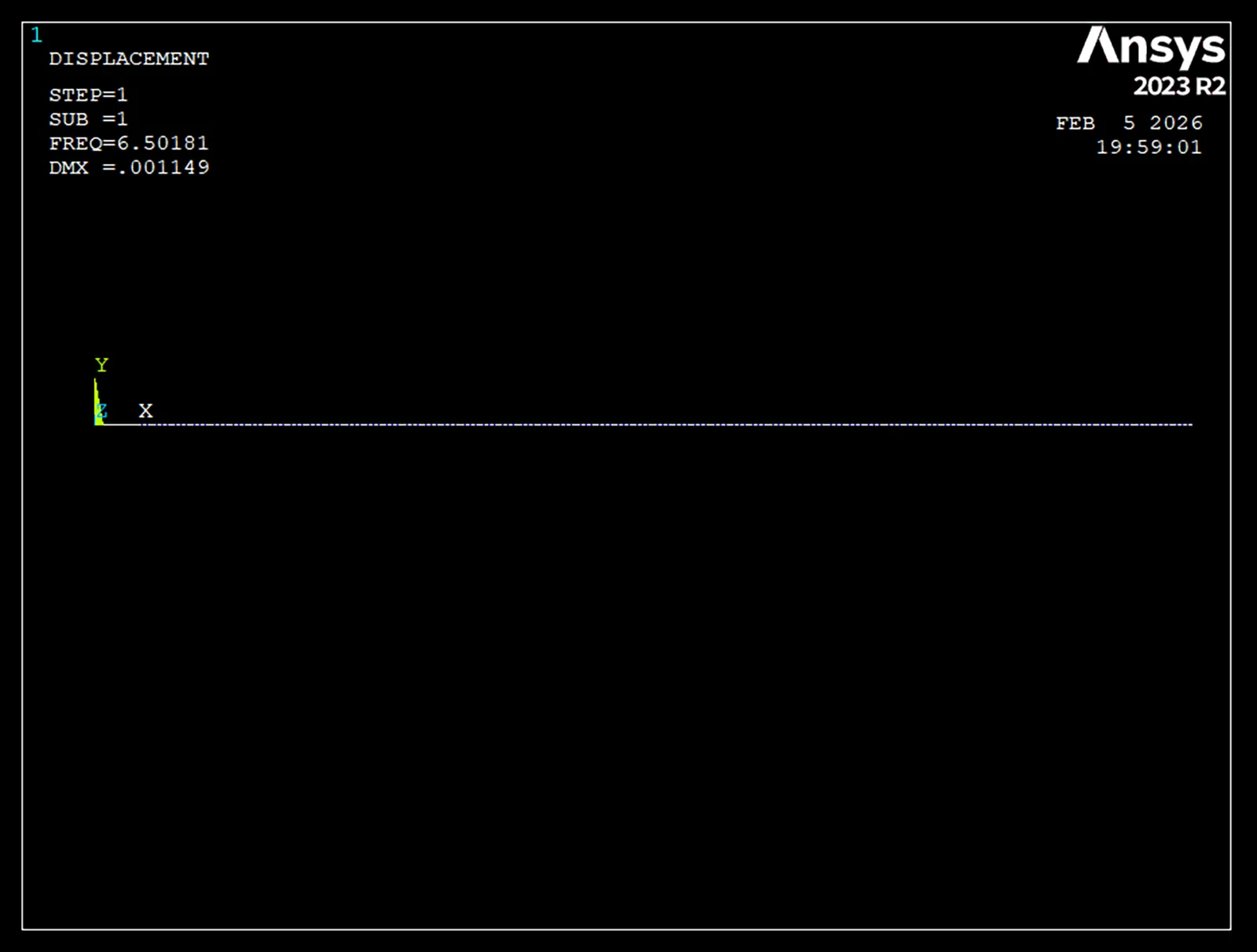
Finite element model of one half (symmetric) of the pilot infrastructure (in Kythira) using ANSYS APDL parametric scripting, showing a good fit with the first vibration frequency measured punctually in-situ.

For the Durangaldea bridge, a detailed model of the main span was developed, showing good fit with the first vibration frequency measured in-situ ( Figure 9). For the Kythira bridge, a more simplified model of one half (symmetric) of the infrastructure was created due to the unavailability of detailed engineering drawings ( Figure 10). These FE models serve multiple purposes: i) validation of AI algorithms through generation of synthetic damaged signals; ii) reference for interpreting anomaly detection results; iii) digital twin capabilities for “what-if” analyses; iv) comparison with natural frequencies extracted from measurements;

### Stakeholder engagement and co-creation

Co-creation activities with Multi-Actor Pilots (MAPs) throughout the development process ensured that the platform addresses real-world needs. ^34, 55^ Specific user needs identified through stakeholder interaction emphasised: i) Automatic detection of anomalies in bridge behavior, particularly in response to environmental challenges; ii) User-friendly interfaces accessible to non-technical users; iii) Integration with existing infrastructure management workflows; iv) Cost-effectiveness suitable for deployment in budget-constrained rural settings. The collected feedback shows the potential of the solution to offer a low-cost but efficient system to monitor infrastructure, with adjustments made based on stakeholder input to satisfy specific rural infrastructure needs.

### Platform implementation

**Figure 11.**
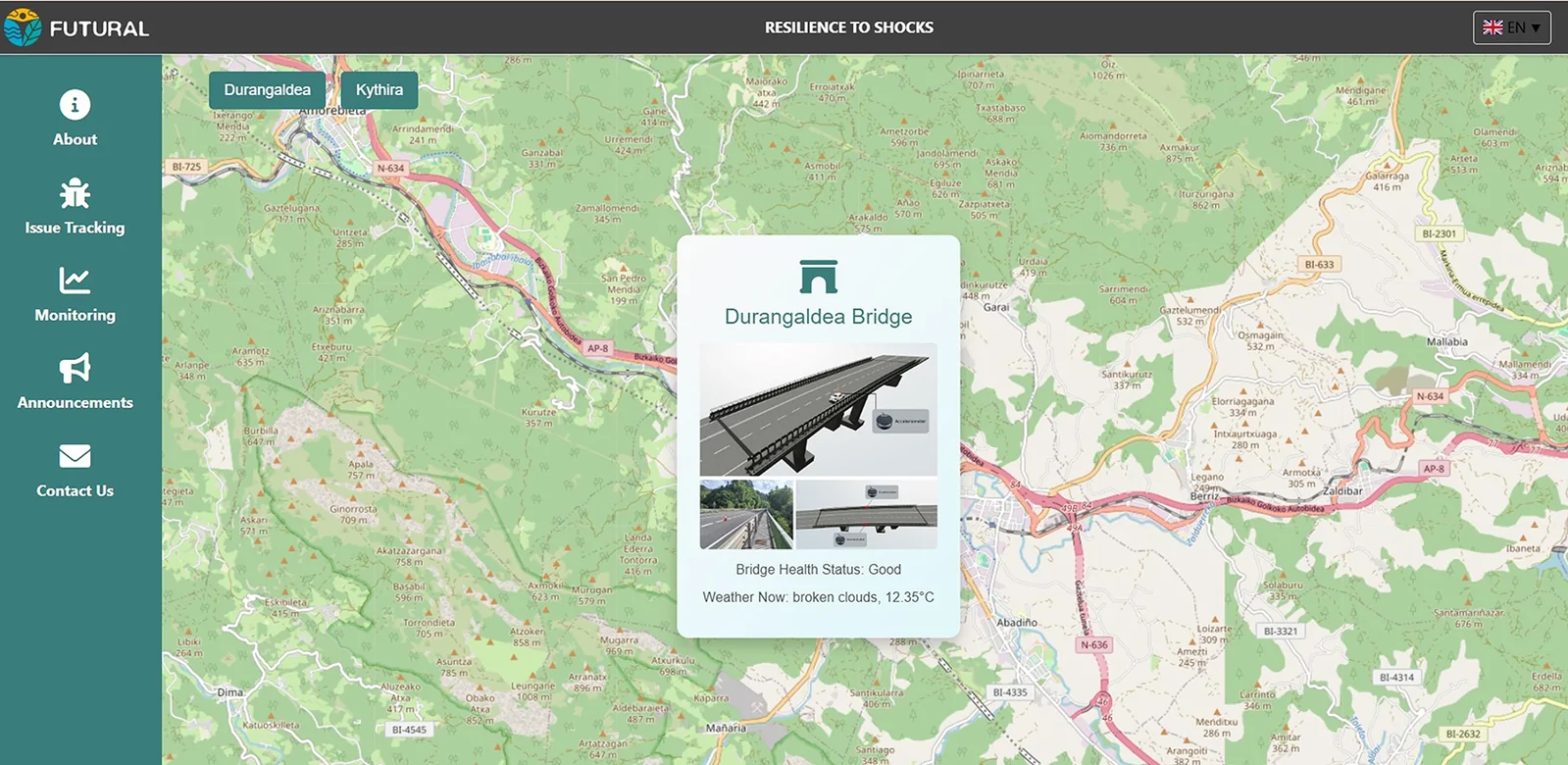
Homepage of the Resilience to Shocks web platform for the Durangaldea Bridge.

**Figure 12.**
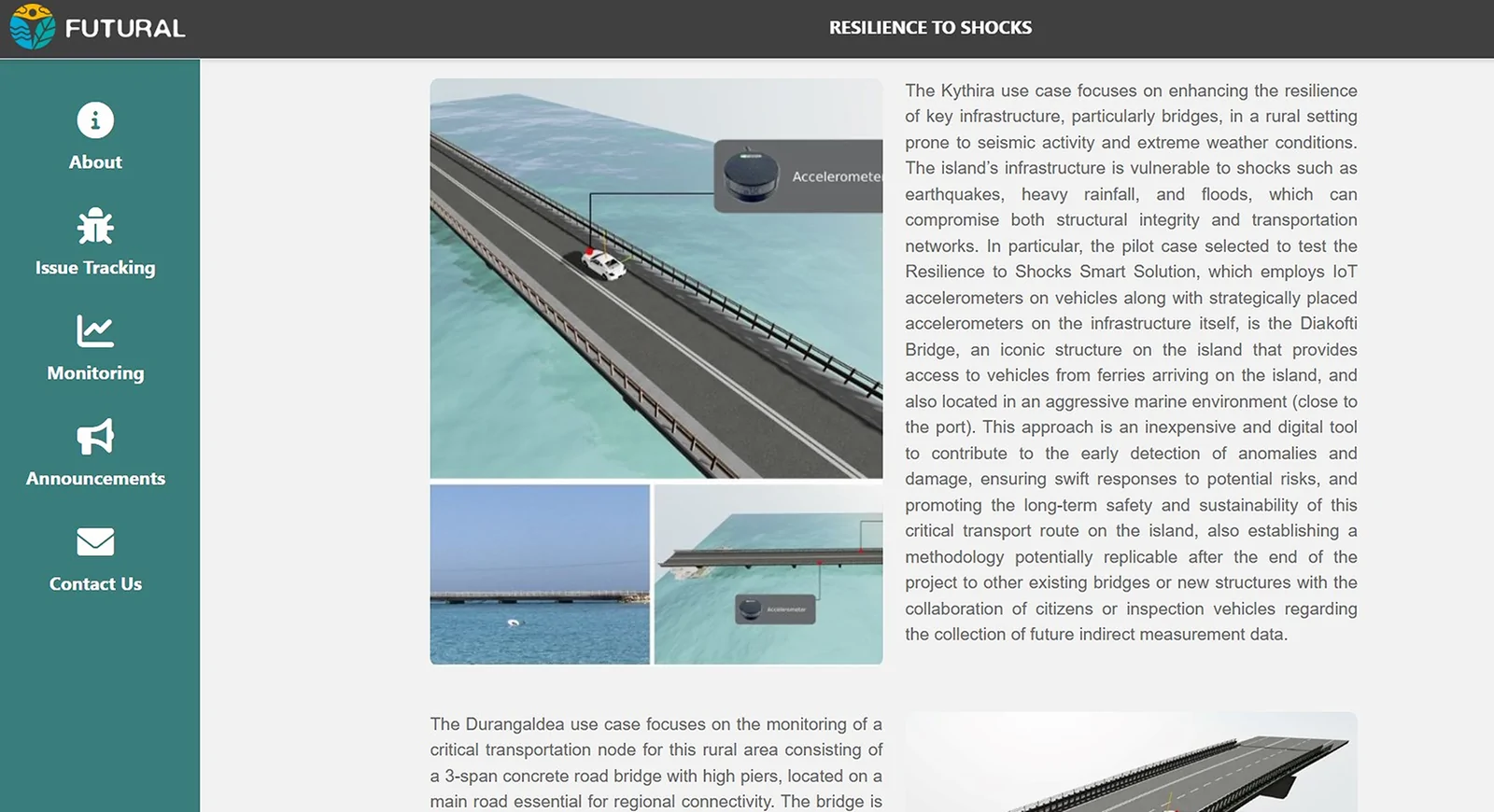
Homepage of the Resilience to Shocks web platform for the description of the Kythira Use case.

The main result is a high-end graphical web tool for the Resilience to Shocks Smart Solution accessible at http://resilience-to-shocks.futural-project.eu/ with multilingual support (English, Greek, and Spanish). ^55^ The platform includes several key functionalities that have been successfully implemented and deployed: i) Homepage with project overview and interactive map; ii) Issue tracking functionality where users (i.e. citizens in the rural regions of Kythira and Durangaldea) can report infrastructure concerns; iii) Infrastructure monitoring dashboard with real-time health status; iv) Historical data visualization; v) Announcements section; vi) Contact form for stakeholder engagement. Figure 11 illustrates the Homepage of the Resilience to Shocks web platform for the Durangaldea Bridge pilot case, showing the interactive map with bridge location, Structural Health Index gauge displaying real-time health status, current weather conditions, and 3D bridge model visualization. Similarly, Figure 12 illustrates the Homepage of the Resilience to Shocks web platform for the Kythira use case, presenting the bridge description panel with Structural Health Index gauge, weather information, prediction status, and interactive 3D model of the Diakofti Bridge in its marine environment.

**Figure 13.**
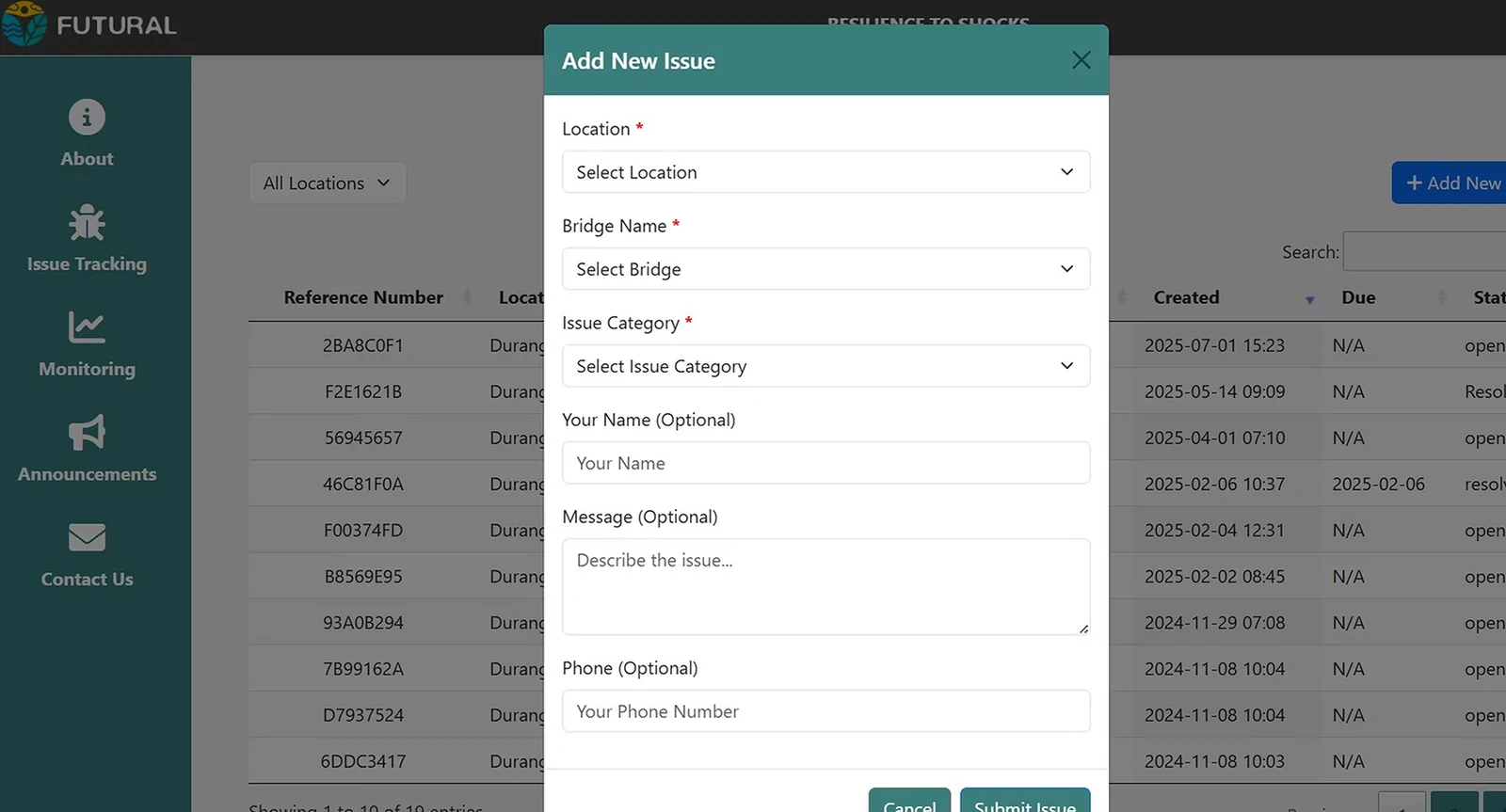
Issue Tracking Page where rural citizens can report issues on the infrastructure network of Durangaldea and Kythira.

Beyond automated sensor-based monitoring, the Resilience to Shocks platform incorporates a participatory dimension through its Issue Tracking module ( Figure 13). This feature empowers local citizens, municipal workers, and regular road users to actively contribute to infrastructure safety by reporting observed concerns directly through the platform. The intuitive submission form allows users to select the location and specific bridge, categorise the type of issue encountered, and provide a detailed description of their observations, all without requiring technical expertise or account registration. Contact information remains optional, lowering barriers to participation. Each submitted issue receives a unique reference number and is tracked through the system with creation dates and resolution status, ensuring transparency and accountability in how reports are handled. This crowdsourced approach to infrastructure monitoring creates a valuable complement to the IoT sensor network. In particular, while automated systems detect structural anomalies through vibration analysis, human observers can identify visual defects, unusual conditions, or contextual factors that sensors cannot capture. The list view displays all reported issues with their current status (opened or resolved), enabling both infrastructure managers and citizens to follow up on concerns. This collaborative model transforms rural communities from passive beneficiaries into active participants in safeguarding their critical transport infrastructure, encouraging a sense of shared responsibility and strengthening the connection between citizens and local authorities.

**Figure 14.**
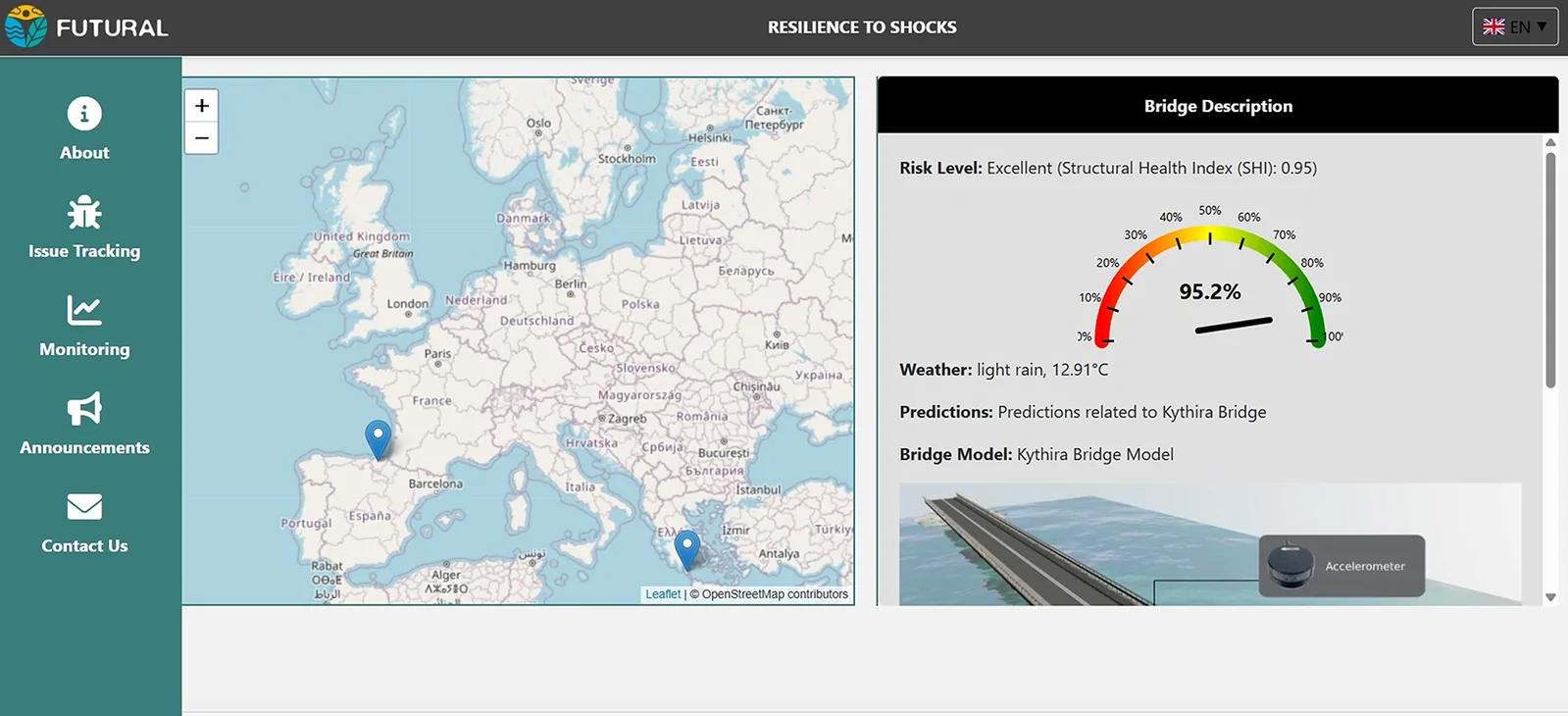
Structural Health Index integration with data infrastructure showing the real-time health status for the Kythira bridge model.

**Figure 15.**
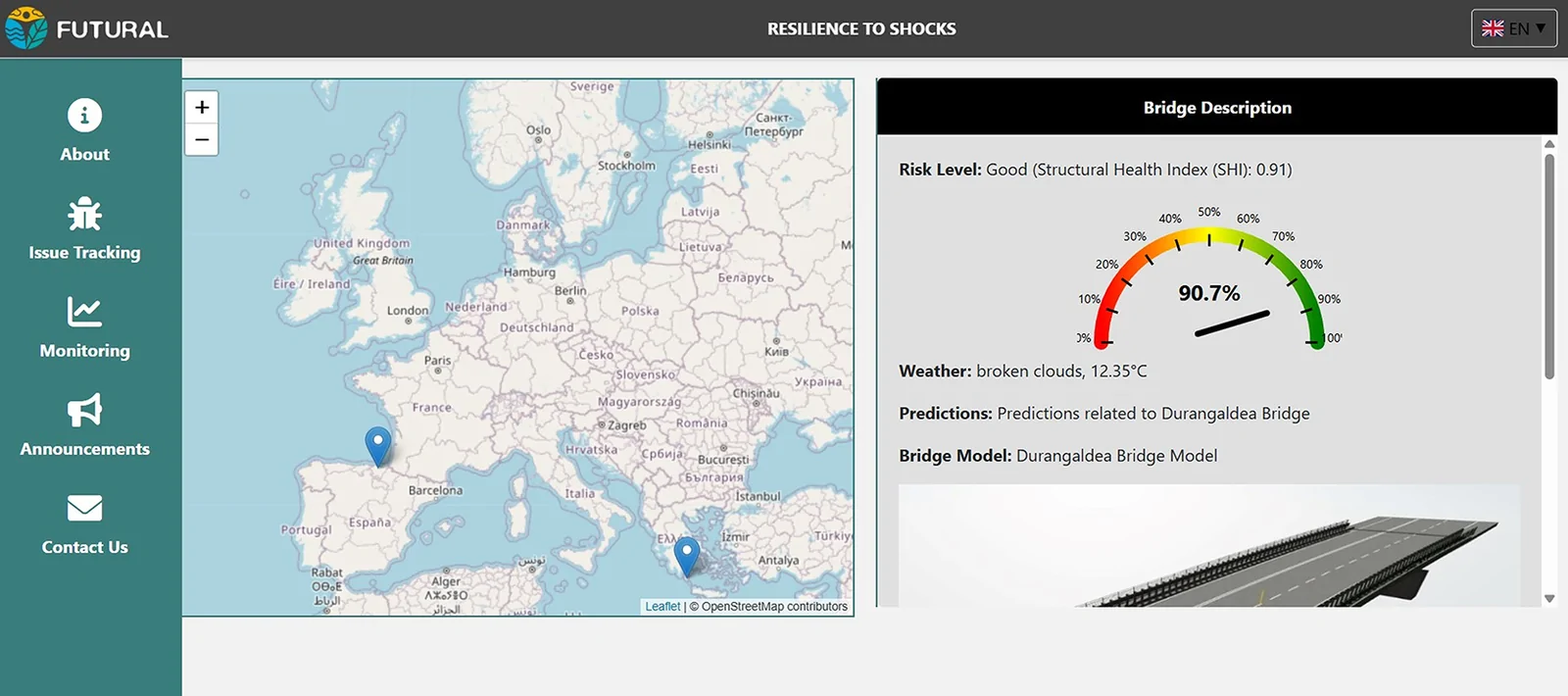
Structural Health Index integration with data infrastructure showing the real-time health status for the Durangaldea bridge model.

The Structural Health Index (SHI) is a comprehensive metric used to assess the overall condition of bridge infrastructure, ranging from 0 to 1, where 1 represents optimal structural integrity. This index is derived from real-time sensor data collected through IoT-based monitoring systems, including accelerometers and vibration sensors that continuously measure the bridge's dynamic response to environmental and traffic loads. The SHI value is classified into five distinct health categories: Excellent (SHI ≥ 0.95) indicates the structure is performing at peak condition with no detectable anomalies: i) Good (SHI ≥ 0.85) reflects a healthy structure with minimal deviation from baseline parameters; ii) Fair (SHI ≥ 0.70) suggests the structure remains serviceable but may require routine monitoring or minor maintenance; iii) Poor (SHI ≥ 0.50) signals notable deterioration that warrants detailed inspection and potential remediation; and Critical (SHI < 0.50) indicates significant structural concerns requiring immediate attention and possible load restrictions. This classification system enables infrastructure managers and citizens to quickly understand the health status of monitored bridges, facilitating proactive maintenance decisions and ensuring public safety. Figure 14 illustrates the implementation of the Structural Health Index within the Resilience to Shocks platform for the Kythira bridge, demonstrating how real-time sensor data is processed and visualized to provide infrastructure managers with an immediate understanding of the bridge's current health status according to the five-tier classification system. Figure 15 presents the corresponding Structural Health Index dashboard for the Durangaldea bridge pilot case, showcasing the integration between the IoT sensor network, cloud-based data processing, and the user-facing interface that translates complex vibration measurements into actionable health categories ranging from Excellent to Critical.

The FUTURAPP application was successfully tested in both pilot locations. In Durangaldea, TECNALIA conducted data collection campaigns with vehicles crossing the bridge under various operational conditions. Testing of the system in Kythira demonstrated usability differences between sensor options. While a development kit capable of deploying on-chip AI was initially considered, it was discarded due to poor usability and power supply difficulties. The FUTURAPP solution proved highly usable, requiring minimal instructions for operation. ^55^ A key design principle of the Resilience to Shocks platform is accessibility for non-technical users, including rural citizens and local authorities who may not have engineering backgrounds. The Structural Health Index (SHI) is displayed using an intuitive gauge meter that resembles a familiar car speedometer. This color-coded dial transitions from red (critical) through yellow (fair) to green (excellent), allowing users to instantly grasp the bridge's health status at a glance without needing to interpret complex numerical data or technical graphs. The current reading of 96.2% is prominently displayed in the green zone, immediately communicating that the Kythira Bridge is in excellent condition. This simple visual metaphor is universally understood across cultures and education levels, and transforms complex sensor data and AI-driven analysis into actionable information that any citizen can comprehend. Complementing this gauge, the platform also displays real-time weather conditions and provides access to 3D bridge models, creating a comprehensive yet user-friendly dashboard that empowers rural communities to stay informed about the safety of their critical infrastructure.

## Scientific contributions and innovations

This work presents several key innovations in the field of structural health monitoring for rural infrastructure: i) **Crowdsensing Paradigm**: The approach represents a paradigm shift from traditional monitoring where most monitoring is by human inspection or fixed instrumentation, toward a future where monitoring can be indirect, utilizing passing vehicles. ^54^ This significantly reduces the per-bridge cost of monitoring, enabling widespread deployment in underserved regions. ii) **Integration of Low-Cost IoT with Machine Learning**: The combination of affordable sensors (USB-C accelerometers with smartphones) with autoencoder-based anomaly detection simplifies the system architecture while maintaining detection capability. This represents a technological breakthrough in making advanced SHM accessible to regions with limited resources. ^57^ iii) **Unsupervised Learning Approach**: The use of autoencoders for unsupervised anomaly detection provides a robust approach that does not require labeled data from damaged conditions. This is particularly important for practical applications where damaged data is typically unavailable. iv) **Multi-Platform Integration**: The architecture integrating mobile application, cloud processing, FE modeling, and web visualization provides a comprehensive solution addressing the full workflow from data collection to decision support.

Compared to existing drive-by monitoring approaches, the Resilience to Shocks platform offers several advantages: i) **Usability**: The FUTURAPP application was designed for non-expert users, whereas many research approaches require specialized equipment and expertise. ii) **Cost-Effectiveness **: Using smartphones with USB-C sensors rather than dedicated data acquisition systems significantly reduces deployment costs. ^53^ iii) **Scalability**: The cloud-based architecture supports multiple bridges and users without requiring local infrastructure installation. However, limitations remain compared to direct SHM systems: i) **Detection Sensitivity**: Indirect monitoring is inherently less sensitive than direct monitoring for detecting small localised damage. ^33^ ii) **Data Quality Dependence**: Detection performance depends on road surface conditions and vehicle properties, introducing variability not present in fixed sensor systems. iii) **Training Requirements**: Collecting sufficient baseline data for algorithm training requires time and repeated vehicle passages.

The platform has significant practical implications for rural infrastructure management: i) **Resource Efficiency**: By enabling monitoring with low-budget IOT sensor installations, the approach allows infrastructure managers to allocate limited budgets more effectively, potentially monitoring multiple bridges at the cost traditionally required for one; ii) **Rapid Assessment**: The ability to assess bridge conditions through vehicle passages enables rapid post-event assessment following earthquakes, floods, or other shock events, supporting decision-making on road closures and emergency routing; iii) **Citizen Engagement**: The crowdsensing approach can leverage citizen participation through regular vehicle users willing to carry sensors, creating community engagement in infrastructure safety; iv) **Scalable Deployment**: The methodology established through the pilot cases is potentially replicable across the European rural transport network with minimal modifications.

Several limitations should be acknowledged: i) **Signal Duration**: Short vehicle crossing times limit frequency resolution and the amount of information per passage. GPS-triggered automatic measurement start/stop is being developed to optimise signal capture. ii) **Environmental Influences**: While the autoencoder learns to accommodate some environmental variability, comprehensive evaluation under diverse conditions (seasonal temperature variations, precipitation) is ongoing. iii) **Training Data Requirements**: Reliable detection requires sufficient baseline data. Currently 30 passages have been used, but collecting more data will improve the reliability of the predictions. ^55^ Some adjustments will be necessary to obtain good results with a larger dataset, such as improvements in the neural network architecture or changes in the thresholds for the reconstruction error metric. iv) **Single-Bridge Training**: Current algorithms are trained per-bridge, requiring individual training campaigns for each monitored structure.

Future work will address: i) Extraction of bridge natural frequencies from indirect measurements for comparison with FE models; ii) Real-time data streaming from FUTURAPP to enable immediate detection during vehicle crossings; iii) Integration of permanent low-cost direct monitoring sensors for enhanced detection capability; iv) Expansion of the training dataset to improve prediction reliability.

## Conclusions

This paper presented the Resilience to Shocks Smart Solution, an innovative crowdsensing platform for indirect structural health monitoring of bridge infrastructure in rural areas, developed within the Horizon Europe FUTURAL project. The platform addresses a critical gap in infrastructure management: the need for affordable, scalable monitoring solutions that can serve regions where traditional instrumentation-based approaches are economically unfeasible The developed system successfully integrates multiple technological components into a cohesive end-to-end solution: i) **Low-cost IoT accelerometers** connected via the FUTURAPP smartphone application, enabling data collection without specialised equipment or extensive training; ii) **Cloud-based data processing infrastructure** that bridges raw sensor data collection with advanced analytics, ensuring secure data transmission and storage through standard IoT protocols; iii) **Autoencoder neural networks** for unsupervised anomaly detection, trained on frequency-domain vehicle acceleration data (2-15 Hz) to identify deviations from healthy structural responses without requiring labeled damaged data; iv) **Finite element modeling** using ANSYS APDL parametric scripting for structural validation, calibrated against in-situ measurements to ensure accurate representation of bridge dynamic behavior; v) **Web-based visualisation and decision support dashboard** featuring intuitive Structural Health Index gauges, interactive 3D BIM models, and citizen engagement tools accessible to non-technical users. The deployment in two pilot cases, a three-span concrete road bridge in Durangaldea, Spain, and the Diakofti Bridge in Kythira, Greece, demonstrated the feasibility and practical utility of the approach for real-world rural infrastructure monitoring under diverse environmental conditions, including areas prone to seismic activity, heavy rainfall, and marine exposure.

Key achievements of this work include: i) **Development and successful field testing** of the FUTURAPP mobile application, which proved highly usable for non-expert operators and demonstrated superior practicality compared to alternative development kits; ii) **Implementation of autoencoder algorithms** capable of distinguishing between healthy and anomalous structural responses using reconstruction error as a damage-sensitive feature, with the 99th percentile threshold effectively classifying normal from abnormal conditions; iii) **Calibration of finite element models** against field measurements obtained from high-end environmental vibration accelerometers, achieving good agreement with measured eigenfrequencies for both pilot bridges; iv) **Deployment of an operational web-based platform** at http://resilience-to-shocks.futural-project.eu/, providing multilingual support and citizen-friendly interfaces including issue tracking capabilities that encourage community participation in infrastructure safety; v) **Introduction of the Structural Health Index (SHI)**, a comprehensive metric ranging from 0 to 1 with five distinct health categories (Excellent, Good, Fair, Poor, Critical), enabling infrastructure managers and citizens to quickly understand bridge conditions through intuitive color-coded gauge visualisations.

The crowdsensing methodology offers significant advantages over traditional instrumentation-based approaches. In terms of **cost**, the use of smartphones with USB-C accelerometers eliminates the need for expensive dedicated data acquisition systems on each bridge. Regarding **scalability**, the cloud-based architecture supports monitoring of multiple bridges across wide geographic areas without requiring local infrastructure installation. With respect to **accessibility**, the user-friendly interfaces and intuitive visualisations make advanced structural health monitoring comprehensible to rural communities with limited technical expertise, transforming citizens from passive beneficiaries into active participants in infrastructure safety. While challenges remain, particularly in detection sensitivity for small localised damage, robustness to environmental and operational variability, and the need for sufficient baseline training data, the platform represents a meaningful step toward the digitalisation of infrastructure management in underserved rural regions. The ongoing collection of additional vehicle passage data will continue to improve algorithm reliability, and future developments including GPS-triggered automatic measurements and real-time data streaming will further enhance the system's capabilities. The methodology and tools developed through this project provide a solid foundation for future expansion and replication across the European rural transport network. The open architecture and standardised protocols facilitate adaptation to different bridge types and monitoring requirements, while the lessons learned from the Durangaldea and Kythira pilot deployments offer valuable guidance for future implementations. By demonstrating that advanced infrastructure monitoring is achievable in resource-constrained settings, this work contributes to the broader objectives of the European Union in promoting smart, connected, and resilient communities, ensuring that rural areas are not left behind in the digital transformation of transport infrastructure management.

## Ethics and consent statement

Ethical approval and consent were not required for this study. The research involved monitoring of public bridge infrastructure using non-invasive sensor technologies and did not involve human participants, personal data collection, animal subjects, or any procedures requiring ethical review. The citizen engagement features of the platform (issue reporting) collect only voluntarily submitted, non-identifiable information, and users are informed that contact details are optional. All infrastructure monitoring activities were conducted with the authorization of the relevant infrastructure owners (Provincial Council of Bizkaia, Spain, and local authorities in Kythira, Greece).

## Declaration of generative AI and AI-assisted technologies in the writing process

During the preparation of this manuscript, the authors used generative AI-based tools, specifically large language models such as ChatGPT (OpenAI), solely to assist with language editing and consistency checks in limited sections. All substantive decisions regarding the design of the review, screening, data extraction, analysis and interpretation were made by the authors. The authors carefully reviewed and edited all AI-assisted text and take full responsibility for the integrity and accuracy of the manuscript’s content.

## Data Availability

The crowdsensing platform is publicly accessible at: http://resilience-to-shocks.futural-project.eu/. The datasets generated and analysed during this study are available under restricted access due to the following reasons: **Restrictions on data access:** The raw acceleration measurement data collected from the pilot bridges in Durangaldea (Spain) and Kythira (Greece) contain location-specific structural response information that, in combination with precise infrastructure coordinates, could potentially be misused to identify structural vulnerabilities. Additionally, some data were collected in collaboration with local authorities (Provincial Council of Bizkaia and Municipality of Kythira) under data sharing agreements that restrict public dissemination without prior authorization. **Institutional review:** The FUTURAL project consortium, in accordance with the project's Data Management Plan approved under Horizon Europe Grant Agreement No. 101060835, determined that full open access to raw structural monitoring data requires authorization from the infrastructure owners and relevant local authorities to ensure responsible use. Source code available from: https://github.com/ntantaroudas/Resilience-2Shocks-Platform.git Archived software available from: https://doi.org/10.5281/zenodo.18338089 License: MIT License The web platform is accessible via standard web browsers on desktop and mobile devices: http://resilience-to-shocks.futural-project.eu/
