# Supplementary material for: A crowdsensing platform for structural health monitoring of rural bridge infrastructure
Source: Open Res Eur. 2026 Sep 22;6:50. Originally published 2026 Feb 16. [Version 2] doi: 10.12688/openreseurope.22915.2 (PMC13612994; doi:10.12688/openreseurope.22915.2)
Supplement: Peer review history

# Reviewer report: Reviewer response for version 2

**DOI:** 10.21956/openreseurope.26094.r81571

**Published:** 2026-09-25

**Authors:** Marco Civera

**Affiliations:** 1 Polytechnic of Turin, Turin, Piedmont, Italy

The report is substantially improved. “Anomalous” is now defined appropriately as the deviation from the learned baseline. Operational variability is discussed as well, even though it is claimed to be mainly controlled by using the same vehicle and approximately constant speed and is not actually handled or compensated for by the algorithm. The new limitations and conclusions clearly acknowledge the absence of damaged-state measurements, and this is the most relevant improvement. The acquisition protocol is much better described and more thoroughly explained, even if some technical details such as windowing, filtering, and detrending could have been more explicitly reported.

 The authors now explicitly acknowledge the absence of false-positive/false-negative validation, and the restricted-access justification is very clearly explained. Finally, the conclusion is appropriately toned down and now describes the work as a feasibility demonstration rather than a validated damage-detection system. Some residual passages, untouched from version 1, may still overstate structural condition assessment and operational readiness, but the text is overall clear in its main aims and achievements. Hence, all things considered, the research article is definitely approvable in its current form and represents a strong contribution as a platform-level feasibility study of an interesting research programme.

Is the study design appropriate and does the work have academic merit?

Partly

Is the work clearly and accurately presented and does it cite the current literature?

Partly

If applicable, is the statistical analysis and its interpretation appropriate?

Partly

Are all the source data underlying the results available to ensure full reproducibility?

No

Are the conclusions drawn adequately supported by the results?

Partly

Are sufficient details of methods and analysis provided to allow replication by others?

Partly

Reviewer Expertise:

Structural Health Monitoring, vibration-based inspection, crowdsensing

I confirm that I have read this submission and believe that I have an appropriate level of expertise to confirm that it is of an acceptable scientific standard.

# Reviewer report: Reviewer response for version 2

**DOI:** 10.21956/openreseurope.26094.r81572

**Published:** 2026-09-24

**Authors:** Yiming Zhang

**Affiliations:** 1 Southeast University School of Civil Engineering (Ringgold ID: 306525), Nanjing, Jiangsu, China

I have no further comments to make.

Is the study design appropriate and does the work have academic merit?

Partly

Is the work clearly and accurately presented and does it cite the current literature?

Partly

If applicable, is the statistical analysis and its interpretation appropriate?

Partly

Are all the source data underlying the results available to ensure full reproducibility?

No

Are the conclusions drawn adequately supported by the results?

Partly

Are sufficient details of methods and analysis provided to allow replication by others?

Partly

Reviewer Expertise:

Structural Health Monitoring, Bridge Engineering, Machine Learning

I confirm that I have read this submission and believe that I have an appropriate level of expertise to confirm that it is of an acceptable scientific standard.

# Reviewer report: Reviewer response for version 1

**DOI:** 10.21956/openreseurope.24777.r70003

**Published:** 2026-03-14

**Authors:** Yiming Zhang

**Affiliations:** 1 Southeast University School of Civil Engineering (Ringgold ID: 306525), Nanjing, Jiangsu, China

This study presents an innovative crowdsensing platform for structural health monitoring of rural bridge infrastructure, addressing the high cost and poor scalability of traditional monitoring methods via vehicle-based indirect measurements, low-cost IoT sensors and autoencoder-based unsupervised anomaly detection. The platform has been validated on two pilot bridges in Spain and Greece, demonstrating notable engineering application value and academic innovation in rural infrastructure digital management. However, there are still a series of issues in the manuscript that need further clarification and resolution.

**1.** This paper fails to adequately incorporate and discuss the latest research literature published in the past two years relevant to its core research direction. It is recommended to cite relevant studies on low-cost structural health monitoring for small and medium-span bridges, such as:

 Refer to reference no. 1, 2 & 3

**2.** The anomaly detection model is trained using only 30 vehicle passages, which is a relatively small dataset for machine learning-based monitoring.

**3.** The paper focuses heavily on system architecture and platform implementation, while the algorithmic validation and damage detection capability are less rigorously demonstrated.

**4.** The training procedure of the autoencoder is not described in sufficient detail. Providing these details would significantly improve the reproducibility of the study.

**5.** The manuscript introduces a Structural Health Index (SHI) ranging from 0 to 1 to represent the condition of the monitored bridges. However, the mathematical formulation and calculation procedure of the SHI are not clearly described.

**6.** The study uses reconstruction error from an autoencoder as the anomaly indicator and defines the anomaly threshold using the 99th percentile of reconstruction errors from the training data. The justification for choosing the 99th percentile threshold should be explained in more detail.

**7.** The conclusions generally reflect the findings presented in the manuscript. The claim that the system can reliably detect structural anomalies should be moderated given the limited dataset and simplified validation cases.

Is the study design appropriate and does the work have academic merit?

Partly

Is the work clearly and accurately presented and does it cite the current literature?

Partly

If applicable, is the statistical analysis and its interpretation appropriate?

Partly

Are all the source data underlying the results available to ensure full reproducibility?

No

Are the conclusions drawn adequately supported by the results?

Partly

Are sufficient details of methods and analysis provided to allow replication by others?

Partly

Reviewer Expertise:

Structural Health Monitoring, Bridge Engineering, Machine Learning

I confirm that I have read this submission and believe that I have an appropriate level of expertise to confirm that it is of an acceptable scientific standard, however I have significant reservations, as outlined above.

## References

[1] : Acoustic signals-based probabilistic fault diagnosis for expansion joints of small and medium bridges using Bayesian ensemble learning. *Engineering Structures* .2026;354: 10.1016/j.engstruct.2026.122379 10.1016/j.engstruct.2026.122379

[2] : A temperature-driven approach for quantitative assessment of strengthening effect of continuous bridges using structural health monitoring data. *Structural Health Monitoring* .2024;23(2) : 10.1177/147592172311818821053-1070 10.1177/14759217231181882

[3] : SGFormer: prototype learning enhanced transformer for multi-sensor anomaly detection in lightweight bridge structural health monitoring systems. *Structural Health Monitoring* .2026; 10.1177/14759217261422450 10.1177/14759217261422450

# Reviewer report: Reviewer response for version 1

**DOI:** 10.21956/openreseurope.24777.r70000

**Published:** 2026-03-12

**Authors:** Imane Bayane

**Affiliations:** 1 KTH-Royal Institute of Technology, Stockholm, Sweden

The manuscript presents an IoT-based system and a user-friendly platform applied to bridge monitoring for anomaly detection and reporting. The system appears to be easily connected through a mobile phone, which could make it accessible for non-expert users. Overall, the manuscript is well written. However, I recommend addressing the following points before INDEXING:

 When referring to AI or AI algorithms, be more specific. For example, do you refer to autoencoders or other specific methods?

 While the references provided are relevant, the literature review is not extensive. Consider including more recent studies

 Define the functions *f* and *g* in the text and explain their roles in the methodology.

 In the autoencoder figure, specify the type of data used as input and what *x* *i* represents

 It would be helpful to add a figure illustrating the different steps of the system architecture, including the types of data collected, how it is processed and stored.

 Can you include more detailed information about the two bridges studied in your text, such as dimensions, year of construction…

 It is not clear what causes the abnormal signals or how they are generated. Clarify this

 Figure 9 could be improved by refining the visualization in ANSYS.

 Figure 10 is not clear and currently shows only one dashed line. Can you improve the figure?

 Explain how the structural index is calculated and provide the relevant equation. Is a subjective index?

 How does the system detect the presence of a vehicle? How does the system handle situations where two vehicles cross the bridge simultaneously?

 The cost is mentioned briefly, but it is not clearly discussed in the main text. The comparison is not fully justified, since the results obtained from the traditional data acquisition system and the proposed system are not equivalent. Provide more details regarding the cost of the proposed system and how it compares with traditional monitoring systems respecting the outcomes of each one.

Is the study design appropriate and does the work have academic merit?

Yes

Is the work clearly and accurately presented and does it cite the current literature?

Yes

If applicable, is the statistical analysis and its interpretation appropriate?

Not applicable

Are all the source data underlying the results available to ensure full reproducibility?

Partly

Are the conclusions drawn adequately supported by the results?

Partly

Are sufficient details of methods and analysis provided to allow replication by others?

Partly

Reviewer Expertise:

Monitoring, Bridge, Anomaly Detection

I confirm that I have read this submission and believe that I have an appropriate level of expertise to confirm that it is of an acceptable scientific standard, however I have significant reservations, as outlined above.

# Reviewer report: Reviewer response for version 1

**DOI:** 10.21956/openreseurope.24777.r69998

**Published:** 2026-03-02

**Authors:** Marco Civera

**Affiliations:** 1 Polytechnic of Turin, Turin, Piedmont, Italy

This research article presents the “Resilience to Shocks Smart Solution” developed in the Horizon Europe FUTURAL project: an end-to-end crowdsensing platform for affordable structural health monitoring (SHM) of rural bridges, using vehicle-based indirect measurements complemented by low-cost on-structure IoT sensing. The system integrates (i) a mobile app (FUTURAPP) connecting a USB-C accelerometer to a smartphone/tablet for acquisition and basic FFT processing, (ii) cloud infrastructure for transmission/storage, (iii) an autoencoder-based unsupervised anomaly detector trained on frequency-domain acceleration magnitudes (2–15 Hz), (iv) finite element (FE) models in ANSYS Mechanical APDL for structural validation/digital-twin use, and (v) a web dashboard with a “Structural Health Index” (SHI) visualization and an issue-reporting module for citizen engagement. The platform is demonstrated on two pilot bridges: Durangaldea (Spain) and the Diakofti Bridge in Kythira (Greece).

 The work is very interesting and well-done. It can definitely provide useful future applications to the field.

 Some minor comments to further improve the presentation of the work:

 (1) The manuscript is generally readable and provides a helpful high-level walkthrough of indirect SHM, crowdsensing concepts, and how the FUTURAL platform components fit together. The literature review is broad, covering recent work up to 2024–2025, which is a strength, even if some of the very most recent works on crowdsensing are missing.

 (2) Some technically important elements are described only qualitatively, which makes it hard to assess accuracy and to understand what exactly is implemented versus planned. Examples include how the SHI is computed from model outputs (not just its categorical thresholds), how the anomaly detector handles operational variability (vehicle type/speed/road roughness), and what constitutes “anomalous” beyond the provided contrast examples

 (3) The academic merit is clear as a systems/translation contribution: integrating low-cost sensing, cloud workflows, unsupervised learning, FE modelling, and citizen-facing interfaces into a deployable platform for rural bridges is valuable, and the pilot deployments add credibility. However, the validation design for the anomaly detection component is currently weak if interpreted as “damage detection.” Actual proof of sensitivity to realistic deterioration/damage on the bridges seems a bit limited. This point should be better stated in the text.

 (4) Core elements are well outlined, but full replication would require additional specifics that are not currently provided in the manuscript, such as acquisition protocol (sampling rate, record length, triggering, axis/orientation, mounting location in vehicle, vehicle type/speed distribution, road roughness handling); signal pre-processing details (windowing, FFT resolution/normalization steps, how the 650-length input vector is formed, filtering/detrending); ML training details (train/validation split, optimizer, learning rate, epochs, batch size, early stopping, random seeds, framework versions); and thresholding rationale and procedure (how threshold is selected, expected false alarm rate on unseen “healthy” data).

 (5) The work uses a percentile rule (99th percentile of training reconstruction errors) as an operational decision threshold. This is a reasonable starting point for one-class/anomaly detection, but the paper does not quantify performance in terms of false positives/false negatives. Without such analysis, the interpretation that the model “distinguishes healthy vs anomalous bridge condition” is not yet fully demonstrated in a SHM sense. Please further highlight this point when discussing this work’s limitations.

 (6) The code is available via GitHub/Zenodo, which is positive, even if it is not sufficient for full reproducibility of the reported empirical results without at least some accessible data. The underlying datasets are explicitly under restricted access, with motivations clearly explained and understandable; thus, if the funding agency allows this option, to this Reviewer’s opinion, it is feasible to keep the data from being publicly available. Potentially, artificially generated mock data can be added, only for testing out the provided codes

 (7) The conclusions about platform integration, usability intentions, and the feasibility of a low-cost workflow are broadly consistent with the described deployment. However, stronger claims around anomaly detection as indicative of bridge condition/damage and replication “across European transport networks” are not fully supported by the limited validation described in the paper. These claims should be rephrased and partially toned down.

Is the study design appropriate and does the work have academic merit?

Partly

Is the work clearly and accurately presented and does it cite the current literature?

Partly

If applicable, is the statistical analysis and its interpretation appropriate?

Partly

Are all the source data underlying the results available to ensure full reproducibility?

No

Are the conclusions drawn adequately supported by the results?

Partly

Are sufficient details of methods and analysis provided to allow replication by others?

Partly

Reviewer Expertise:

Structural Health Monitoring, vibration-based inspection, crowdsensing

I confirm that I have read this submission and believe that I have an appropriate level of expertise to confirm that it is of an acceptable scientific standard, however I have significant reservations, as outlined above.
